# Meloxicam protects against muscle and metabolic decline in ageing through Cdon restoration and oxidative stress modulation

**DOI:** 10.1038/s12276-026-01804-1

**Published:** 2026-08-12

**Authors:** Ju-Hyeon Bae, Hyun-Kyung So, Yideul Jeong, Jeongmin Park, June Kim, Tae Young Kim, Young-Eun Leem, Tuan Anh Vuong, Jiwoong Jang, Moongi Ji, Hyon-Seung Yi, Il-Young Kim, Man-Jeong Paik, Sang-Jin Lee, Gyu-Un Bae, Jong-Sun Kang

**Affiliations:** 1 Department of Molecular Cell Biology, Sungkyunkwan University School of Medicine, Suwon, 16419 Republic of Korea; 2 Research Institute of Aging Related Disease, AniMusCure Inc., Suwon, 16419 Republic of Korea; 3 Department of Metabiohealth, Sungkyunkwan University, Suwon, 16419 Republic of Korea; 4 Drug Information Research Institute, Muscle Physiome Research Center, College of Pharmacy, Sookmyung Women’s University, Seoul, 04310 Republic of Korea; 5 Department of Molecular Medicine, Lee Gil Ya Cancer and Diabetes Institute Gachon University School of Medicine, Incheon, 21999 Republic of Korea; 6 College of Pharmacy, Sunchon National University, Suncheon, 57922 Republic of Korea; 7 Department of Medical Science, Chungnam National University, Daejeon, 34134 Korea

**Keywords:** Molecular biology, Cell biology

## Abstract

Sarcopenia is characterized by progressive decline in skeletal muscle mass and function, driven in part by impaired mitochondrial homeostasis and redox imbalance. However, the upstream regulators that integrate metabolic resilience with muscle integrity during ageing remain poorly defined. Here, we identify the cell adhesion molecule Cdon as a conserved determinant of adult muscle maintenance whose expression declines with human aging, sarcopenia and muscle-wasting disease. Reduced Cdon expression was associated with transcriptional atrophy signatures, metabolic insufficiency and reduced myofibre size. Using a Cdon promoter-based screen, we identified meloxicam (Mcam) as a small molecule inducer of Cdon that enhances myogenic differentiation, increases muscle mass and function in young mice and mitigates age-related muscle atrophy. Mcam treatment also improved neuromuscular conduction and systemic metabolic parameters, including blood glucose regulation and hepatic lipid accumulation. In aged muscle, Mcam normalized cysteine accumulation, restored redox balance, improved mitochondrial metabolism and reduced oxidative stress. Mechanistically, Mcam activated Ampk signalling to preserve Cdon expression under oxidative challenge and support antioxidant and mitochondrial quality control pathways. Together, these findings identify Cdon decline as a hallmark of muscle ageing and demonstrate that Mcam preserves muscle integrity by reestablishing redox and metabolic homeostasis through Ampk-dependent maintenance of Cdon.

**Figure:**
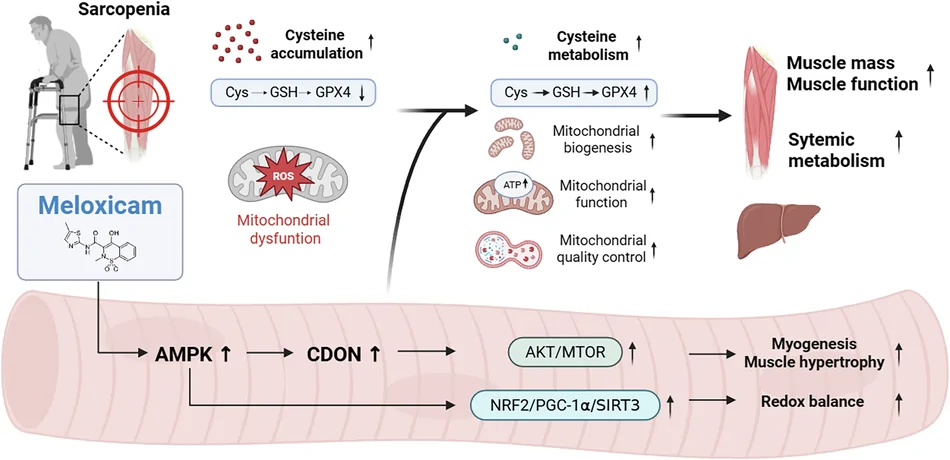
Age-related decline of Cdon is a key molecular feature of skeletal muscle dysfunction. In this study, meloxicam restored Cdon expression through Ampk activation, thereby preserving muscle integrity and functional capacity in aged muscle. Mechanistically, meloxicam normalized cysteine metabolism and elevates Gpx4 expression, improving glutathione buffering and suppressing excessive reactive oxygen species accumulation. Activation of the Ampk–Cdon axis was associated with maintenance of mitochondrial homeostasis and redox balance. Together, these findings identify Cdon as a potential therapeutic target for age-associated skeletal muscle degeneration.

Age-related decline of Cdon is a key molecular feature of skeletal muscle dysfunction. In this study, meloxicam restored Cdon expression through Ampk activation, thereby preserving muscle integrity and functional capacity in aged muscle. Mechanistically, meloxicam normalized cysteine metabolism and elevates Gpx4 expression, improving glutathione buffering and suppressing excessive reactive oxygen species accumulation. Activation of the Ampk–Cdon axis was associated with maintenance of mitochondrial homeostasis and redox balance. Together, these findings identify Cdon as a potential therapeutic target for age-associated skeletal muscle degeneration.

## Meloxicam Restores Muscle Function by Targeting Cdon Levels

Sarcopenia, the age-related loss of muscle mass and strength, significantly impacts older adults’ health, yet its regulatory mechanisms remain elusive. This study identifies Cdon, a protein whose expression declines with age, as a crucial factor in muscle maintenance. Researchers discovered meloxicam (Mcam), a compound that induces Cdon expression, through a chemical screen. Mcam enhances myogenesis, muscle mass, and function in both young and aged mice by activating AMP-activated protein kinase (Ampk), which supports mitochondrial function and redox balance. Mcam treatment improved mitochondrial bioenergetics, reduced oxidative stress and normalized cysteine metabolism, crucial for maintaining muscle integrity. These findings suggest that targeting the Ampk-Cdon pathway could offer therapeutic potential for combating age-related muscle degeneration, positioning Cdon as a promising target for future interventions in sarcopenia and related muscle diseases.

This summary was initially drafted using artificial intelligence, then revised and fact-checked by the author.

## Introduction

Sarcopenia, the age-associated loss of skeletal muscle mass and strength, contributes substantially to frailty, reduced mobility, metabolic dysfunction and increased mortality in older adults^1,2^. Although several characteristic features of muscle ageing have been described, including mitochondrial decline, oxidative stress, impaired proteostasis, metabolic inflexibility and neuromuscular degeneration^3–5^, the upstream regulatory mechanisms that integrate these processes have not been fully defined, limiting the development of targeted therapeutics.

Mitochondrial dysfunction is recognized as a central driver of muscle ageing^6^. Reductions in oxidative phosphorylation, ATP depletion and accumulation of reactive oxygen species (ROS) disrupt cellular homeostasis and compromise contractile function^7,8^. Oxidative stress also impairs protein synthesis, alters organelle quality control and promotes neuromuscular instability^9–11^. Although these interrelated defects collectively contribute to functional decline, the signalling networks that maintain redox balance and metabolic resilience during ageing remain incompletely understood.

Ampk coordinates essential metabolic and antioxidant responses that support mitochondrial homeostasis and redox balance^12^. In ageing muscle, Ampk signalling appears abnormally modulated, with a reduced response to acute energetic demand yet sustained activation under basal stress, which can act as a form of dysregulation^13^. Although such persistent activation may shift the balance towards catabolic dominance by suppressing Akt/mTOR-dependent anabolic signalling and contributing to muscle wasting, evidence also indicates that restoring a physiological Ampk response, via exercise or context-appropriate activation, can improve mitochondrial quality control, enhance stress resilience and mitigate age-related muscle decline^14–16^.

Cell adhesion molecule-related/downregulated by oncogenes (Cdon) is a multifunctional membrane-associated protein known to regulate myogenic differentiation, satellite cell function and neuromuscular junction stability^17–20^. Cdon expression is inducible under nutrient limitation and energetic stress, suggesting potential involvement in adaptive programs that link metabolic status to muscle maintenance^19,21^. Despite its established developmental role, the function of Cdon in adult muscle homeostasis and its relevance to ageing remain unknown.

In this study, we demonstrate that Cdon expression declines in human ageing, sarcopenia and multiple muscle disease states, and that reduced Cdon is associated with transcriptional signatures of atrophy, metabolic insufficiency and reduced myofibre size. We identify meloxicam (Mcam) through a Cdon reporter-based chemical screen as a pharmacological Cdon inducer that enhances myogenesis, improves muscle mass and function, and preserves neuromuscular conduction in young and aged mice. Mechanistically, we show that Mcam activates Ampk to maintain Cdon expression under oxidative stress, normalizes cysteine-associated redox imbalance, improves mitochondrial bioenergetics and reduces oxidative damage. These findings establish Cdon decline as a hallmark of muscle ageing and identify a pharmacological approach that restores a redox–metabolic axis central to muscle maintenance.

## Materials and methods

### Animal study

Male wild type C57BL/6 J mice were used in this study and maintained at 23 °C under a 12:12 h light–dark cycle with free access to food and water. Mcam (MedChemExpress, HY-B0261) was dissolved in DMSO to prepare a 100-mM stock solution, which was then diluted in an oral dosing vehicle (10% DMSO, 40% PEG300, 5% Tween 80 and normal saline) for mouse administration. To assess the effect of Mcam, mice were orally administered 0.02 mg/kg daily for the duration indicated in the figures. Treadmill running tests were performed using a Columbus Exer-6M treadmill. Mice were acclimatized by running at 7 m/min for 5 min daily for 5 days, followed by a test at 8 m/min on a 10% incline until exhaustion. Grip strength was measured using a grip strength meter (Bioseb). Forelimb grip strength was measured three times per mouse, recording the maximal force when the forelimb released the grid. Electromyographic (EMG) responses were assessed using the RS-EMG-SA-STIM Small Animal EMG System (iWorx Systems). Prior to sacrifice, compound muscle action potentials were recorded from the flexor digitorum brevis muscle following sciatic nerve stimulation prior to sacrifice. At the time of sacrifice, serum was collected for the determination of TG (Elabscience, E-BC-K261-S) and free fatty acids (Invitrogen, EEA017). Blood glucose levels were measured from tail blood using Lifespan glucose monitors after a 16-h fast with free access to water. All animal experiments were approved by the Institutional Animal Care and Research Advisory Committee at Sungkyunkwan University school of Medicine (SUSM) and complied with the regulations of the institutional ethics committee (SKKUIACUC2025-10-47-1).

### Cell culture

C2C12 cell culture was performed as previously described^22,23^. C2C12 myoblasts were cultured in growth medium composed of high-glucose Dulbecco’s Modified Eagle Medium (DMEM; Gibco, 12100-061) supplemented with 15% fetal bovine serum (Gibco, 16000-044), 1X penicillin/streptomycin (Welgene, LS202-02) at 37 °C in 5% CO₂. To induce differentiation of C2C12 myoblasts, cells at near confluence changed growth medium into a differentiation medium composed of DMEM containing 2% horse serum (Gibco, 26050-088) and 1X penicillin/streptomycin. Human skeletal muscle-derived cells (skMDCs) from a 17-year-old male and a 68-year-old male were obtained from Cook Myosite and cultured as previously described^1^. Proliferation and differentiation of skMDCs into myotubes were carried out according to the instructions provided by Cook Myosite.

For high-throughput screening, a 2-kb fragment of the mouse Cdon promoter was cloned into the pGL4 luciferase reporter vector and verified by sequencing. C2C12 myoblasts were transfected with the Cdon promoter-luciferase construct and, 24 h post-transfection, induced to differentiate while simultaneously treated with compounds from a 2,485-member FDA-approved library (MCE). Luciferase activity was then assessed using the Dual-Glo assay.

To assess the effects of Mcam, C2C12 cells were treated with 100 nM Mcam, and 1 mM L-cysteine (Sigma-Aldrich, 168149) prepared in 5% hydrochloric acid was used to evaluate cellular stress. Oxidative stress was induced by treating cells with 1 μM sodium arsenate (SA; Sigma-Aldrich, S7400), and the Ampk inhibitor Compound C (Com C; Calbiochem, 171261-1MGCN) was applied at 1 μM. Lipid peroxidation was analysed using a Lipid Peroxidation Assay Kit (Abcam, ab118970) according to the manufacturer’s instructions. For cell signalling analyses, near-confluent C2C12 cells were starved in differentiation medium for 24 h before Mcam treatment, and measurements were performed at the indicated time points.

### Histology and immunostaining

Histology and immunostaining were performed as previously described^22,24^. Briefly, tibialis anterior (TA) and extensor digitorum longus (EDL) muscles were dissected and embedded in cryomolds. The frozen tissues were stored at −80 °C and sectioned at 10 μm for cryosectioning. For paraffin sections, hearts, kidneys and livers were fixed in 4% (w/v) paraformaldehyde (PFA; Biosesang, P2031) and embedded in paraffin. Paraffin-embedded tissues were cut into 4-μm sections and subjected to haematoxylin and eosin staining. Fibrosis was evaluated using the Picro Sirius Red Stain Kit (Abcam, ab150681) according to the manufacturer’s instructions. Glycerol-3-phosphate dehydrogenase (GPDH) and nicotinamide adenine dinucleotide tetrazolium reductase (NADH-TR) staining were performed on freshly cryo-sectioned TA and EDL muscles as previously described^22^. Cytochrome c oxidase/succinate dehydrogenase (COX-SDH) staining was performed on EDL muscles using the COX-SDH Double Histochemistry Stain Kit (VitroVivo Biotech, VB-3022) according to the manufacturer’s instructions. Positive areas for GPDH, NADH-TR and COX–SDH staining were quantified using the colour threshold function in ImageJ. The colour space was set to Lab, and thresholding was applied to the L* channel with ranges of 0–100 for GPDH, 0–81 for NADH-TR and 0–38 for COX–SDH. Images used for quantification were obtained under identical intensity and gain settings. For visualization purposes, representative images corresponding to each staining from Veh- and Mcam-treated groups were adjusted to the same brightness level.

For immunohistochemistry, cryosections were fixed in 4% PFA and subjected to antigen retrieval by boiling in pH 6.0 sodium citrate buffer for 10 min. Sections were then blocked for 1 h in blocking buffer containing 5% goat serum (Gibco, 16210-072), 0.1% bovine serum albumin (BSA, Bovogen, BSAS) and phosphate-buffered saline (PBS, pH 7.4), followed by overnight incubation with primary antibodies at 4 °C. Secondary antibody goat anti-mouse IgG Alexa 488 (Invitrogen, A-11001) and goat anti-rabbit IgG Alexa 555 (Invitrogen, A11008) were used for 1-h incubation at room temperature. For staining damaged myofibres using IgM as a marker, TA muscle sections were fixed in 4% PFA, blocked 30 min at room temperature and incubated overnight at 4 °C with Alexa Fluor 488 goat anti-mouse IgM (Invitrogen, A-21042). Immunocytochemistry was performed as for immunohistochemistry, but without the antigen retrieval step. The primary antibodies used are listed in Supplementary Table 1. Images were captured by using Biotek Cytation C10 (Agilent) Confocal Imaging Reader (Agilent), LSM-710 confocal microscope (Zeiss) or LSM 980 confocal microscope (Zeiss). Images were subsequently analysed using Fiji software.

### 5-bromo-2’-deoxyuridine incorporation and cytotoxicity test

5-bromo-2’-deoxyuridine (BrdU) incorporation was performed as previously described^25^. Briefly, cells were incubated with 10 μM BrdU (Sigma-Aldrich, B5002) for 10–15 min, fixed/permeabilized with 2 N hydrochloric acid, neutralized with 0.1 M borate buffer (pH 8.5). Cells were stained with anti-BrdU antibody (Chemicon, MAB3222) overnight at 4 °C, followed by incubation with goat anti-mouse IgG Alexa Fluor 488 antibody at room temperature for 1 h. Images were acquired using a Nikon ECLIPSE TE-2000U microscope. Quantification of images was performed using NIS-Elements F (Nikon) and ImageJ software. Cytotoxicity was assessed using an MTT incorporation assay. C2C12 cells were exposed to Mcam or L-cysteine for 24 h, followed by incubation with 0.5 mg/ml MTT (Glentham Life Science) for 2 h. Formazan crystals were then dissolved in DMSO, and the absorbance was read at 570 nm.

### Reactive oxygen species detection, mitochondria functional analysis and colorimetric assay

To analyse superoxide production using immunofluorescence, freshly cryosectioned TA muscles were rinsed in PBS (pH 7.4) for 5 min to remove optimal cutting temperature, then incubated with 5 µM dihydroethidium (MedChemExpress, HY-D0079) at 37 °C for 1 h. Sections were washed three times with PBS (pH 7.4) and imaged using a confocal microscope. Cellular mitochondrial ROS production and mitochondrial membrane potential were assessed using 1 µM MitoSOX Red (Invitrogen, M36008) and 100 nM tetramethylrhodamine, methyl ester (Invitrogen, I34361), respectively, according to the manufacturer’s instructions. Intracellular cysteine levels and glutathione (GSH)/glutathione disulfide (GSSG) ratios were analysed using a cysteine colorimetric assay kit (Elabscience, E-BC-K352-M) and an EZ-GSH/GSSG assay kit (DoGenBio, DG-GLU200), respectively, according to the manufacturer’s instructions

The oxygen consumption rate (OCR) and extracellular acidification rate (ECAR) were determined using a Seahorse XF HS Mini Analyzer (Agilent) with the Seahorse Mito Stress Test Kit (Agilent, 103015-100), following the manufacturer’s instructions. Briefly, C2C12 cells were seeded at 3 × 10⁴ cells per well in Seahorse plates for 24 h. One hour before measurement, cells were incubated in Seahorse DMEM (Agilent, 103575-100) supplemented with 1 mM sodium pyruvate (Agilent, 103578-100), 2 mM glutamine (Agilent, 103579-100) and 10 mM glucose (Agilent, 103577-100) at 37 °C without CO₂. To evaluate mitochondrial stress response, cells were sequentially exposed to 1.5 μM oligomycin, 3 μM FCCP and 0.5 μM rotenone/antimycin A (Rot/AA).

### Protein analysis

Immunoblotting analysis was performed as previously described^22^. Briefly, muscles and liver were snap-frozen in liquid nitrogen, pulverized using a mortar and pestle and homogenized with at least 10 strokes in a homogenizer. Tissues were then lysed in RIPA buffer (Intron, IBS-BR002) with a complete protease inhibitor cocktail (Roche, 11836145001), and protein concentrations were determined using a BCA Protein Assay Kit (Pierce, 23225). Cells were lysed in RIPA buffer on ice for 10 min, followed by protein quantification and sampling in the same manner as tissue samples. Samples were prepared in Laemmli buffer for subsequent analysis. Then, proteins were separated by SDS-PAGE and transferred onto PVDF membranes at 100 mA for 2 h. Membranes were blocked with 5% skimmed milk for 30 min, then incubated overnight at 4 °C with primary antibodies. After washing, membranes were incubated with HRP-conjugated goat anti-mouse IgG (H + L) (Jackson ImmunoResearch, 115-035-003) or HRP-conjugated goat anti-rabbit IgG (H + L) (Invitrogen, G-21234) secondary antibodies for 1 h at room temperature, followed by protein detection using ECL. The primary antibodies used are listed in Supplementary Table 1.

### Quantitative real-time PCR and RNA sequencing

Quantitative real-time PCR (qRT-PCR) was carried out as previously described^22^. Briefly, total RNA was extracted from cell lysates or muscle lysates prepared by pulverizing in liquid nitrogen using the Easy-Blue Total RNA Extraction Kit (Intron, 17061 L). RNA sequencing was performed on total RNA isolated from gastrocnemius (GAS) muscle lysates using the same methods as previously described, with sequencing carried out by E-biogen Corporation using an Illumina NextSeq 500 platform^22^. Genomic DNA was isolated from GAS muscle powder using the Genomic DNA Extraction Kit (Bioneer, K-3032) following the manufacturer’s instructions. Complementary DNA (cDNA) was synthesized using a cDNA synthesis kit (TAKARA, RR037A), followed by qRT-PCR with SYBR Premix Ex Taq (TAKARA, RR420A) on a Thermal Cycler Dice Real Time System (TAKARA) according to the manufacturer’s instructions. The sequences of primers used for qRT-PCR analysis are listed in Supplementary Table 2.

### Gas chromatography–tandem mass spectrometry

Gas chromatography–tandem mass spectrometry (GC–MS/MS) analysis was conducted using a Shimadzu TQ 8040 triple quadrupole system (Shimadzu), equipped with a cross-linked Ultra-2 capillary column (25 m × 0.20 mm internal diameter, 0.11 μm film thickness; 5% phenyl–95% methylpolysiloxane stationary phase) (Agilent). Samples (1.0 μl) were injected into split mode at a 10:1 ratio. The GC oven temperature program was set as follows: for amino acid (AA) analysis, it was initially held at 140 °C for 3 min, followed by a temperature ramp from 140 °C to 300 °C at 8 °C/min; for organic acids (OAs) and fatty acids (FAs), the program started at 100 °C for 2 min and then increased from 100 °C to 300 °C at 10 °C/min. Helium (0.5 ml/min) and argon were employed as the carrier and collision gases, respectively. The ionization process was carried out using electron impact ionization at 70 eV.

### Liquid chromatography–tandem mass spectrometry

The Triple Quadrupole LCMS-8050 system (Shimadzu) was utilized for liquid chromatography–tandem mass spectrometry (LC–MS/MS) analysis. AAs, oxidized glutathione, kynurenine pathway metabolites, nucleosides and nucleotides were separated using an Intrada Amino Acid column (50 mm × 3.0 mm, 3 µm) and an ACQUITY UPLC HSS T3 column (100 mm × 2.1 mm, 1.8 µm). The following system parameters were applied: ionization mode was electrospray ionization, nebulizing gas flow was set to 3.0 l/min, heating gas flow was 10.0 l/min, interface temperature was maintained at 300 °C and desolvation line temperature was 250 °C. The mobile phase for AAs and oxidized glutathione was applied using a gradient elution consisting of acetonitrile (ACN)/tetrahydrofuran (THF)/25 mM ammonium formate in water/formic acid (9/75/16/0.3, *v*/*v*/*v*/*v*) as phase A, and ACN/100 mM ammonium formate in water (20:80, *v*/*v*) as phase B. The mobile phase for kynurenine pathway metabolites, nucleosides and nucleotides was applied using a gradient elution, with phase A consisting of 0.1% formic acid in water and phase B consisting of 0.1% formic acid in ACN.

### Sample preparation for amino acid, organic acid and fatty acid profiling analyses in plasma and muscle tissue from control, ageing and drug treatment groups by gas chromatography–tandem mass spectrometry

For profiling analysis, quadriceps (QUA) muscle tissues were collected and homogenized using an ultrasonicator in water:MeOH (1:1, *v*:*v*) to a concentration of 200 mg/ml. Profiling analysis of AAs, OAs and FAs in plasma and muscle tissues was carried out using ethoxycarbonylation (EOC)/*tert*-butyldimethylsilyl (TBDMS) and methoxime (MO)/TBDMS derivatization, respectively, as outlined in previous studies^26,27^. Briefly, for AA, deproteinization was performed by adding acetonitrile (100 μl) containing norvaline as an internal standard (IS, 0.2 μg) to plasma (30 μl) and muscle tissue samples (6 mg). After centrifugation, the supernatants were diluted in water (1.0 ml) and transferred to dichloromethane (2.0 ml) containing ethyl chloroformate, with the solution adjusted to pH ≥ 12 using 5 M sodium hydroxide. To derivatize amine groups, the two-phase EOC reaction was performed by vortex-mixing for 10 min to generate EOC derivatives. Briefly for OA and FA, deproteinization was performed by adding acetonitrile (100 μl) containing 3,4-dimethoxybenzoic acid (0.1 μg) and pentadecanoic acid (0.1 μg) as ISs to plasma (40 μl) and muscle tissue samples (6 mg). Following centrifugation, 1.0 ml of water was added to the collected supernatant, and then methoxyamine hydrochloride (1 mg) was added. To derivatize carbonyl groups, the solution was brought to pH ≥ 12 with 5.0 M NaOH and incubated at 60 °C for 1 h to generate MO derivatives. Following the EOC/MO reaction, the aqueous phase was acidified to pH ≤ 2 with 10% sulphuric acid, saturated with sodium chloride, and then subjected to sequential extraction using 3.0 ml of diethyl ether and 2.0 ml of ethyl acetate. The extracts, which included triethylamine, were dried under a mild nitrogen stream at 40 °C until complete evaporation occurred.

Subsequently, toluene (10 μl) and *N*-Methyl-*N*-*tert*-butyldimethylsilyl trifluoroacetamide (20 μl) were added to the dried sample and incubated at 60 °C for 1 h to generate the derivatives by TBDMS, which were then analysed using GC–MS/MS.

### Sample preparation for profiling analysis of amino acids, oxidized glutathione, kynurenine pathway metabolites, nucleosides and nucleotides in plasma and muscle tissue from control, ageing and drug treatment groups by liquid chromatography–tandem mass spectrometry

Profiling analysis of AAs and oxidized glutathione in plasma and muscle (QUA) tissue samples was performed by LC–MS/MS without derivatization. Briefly, the deproteinization was performed by adding ACN (60 μl) containing IS (^13^C_1_-phenylalanine; 25 ng, 3,4-dimethylphoibeonzoic acid; 1.25 μg, 3-deazauridine; 2.5 ng) to plasma (30 μl) and muscle tissue samples (6 mg) and mixing for 1 min. After centrifugation, the supernatant was filtered and transferred to an auto-sampler vial for LC–MS/MS injection.

### Star pattern recognition analysis

The levels of AAs, oxidized glutathione, kynurenine pathway intermediates, nucleosides, nucleotides, OAs and FAs were calculated based on standard calibration curves. For comparison, the mean levels of AAs, oxidized glutathione, kynurenine pathway metabolites, nucleosides, nucleotides, OAs and FAs in the ageing and treatment groups were normalized to the corresponding mean levels of the control group. Normalized values were visualized as radial lines extending from a central point, with the endpoints connected to form star-shaped patterns using Microsoft Excel^28^.

### Statistical analysis

Data are presented as mean ± standard deviation or mean ± standard error of the mean, as indicated in the figure legends. Statistical significance between two groups was determined using an unpaired, two-tailed Student’s *t* test. For comparisons among three or more groups, one-way analysis of variance or two-way analysis of variance was performed using GraphPad Prism 8. Statistical significance is indicated as *P < 0.05, **P < 0.01 and ***P < 0.001.

## Results

### Cdon expression is reduced in aged muscle and muscle diseases

**Fig. 1:**
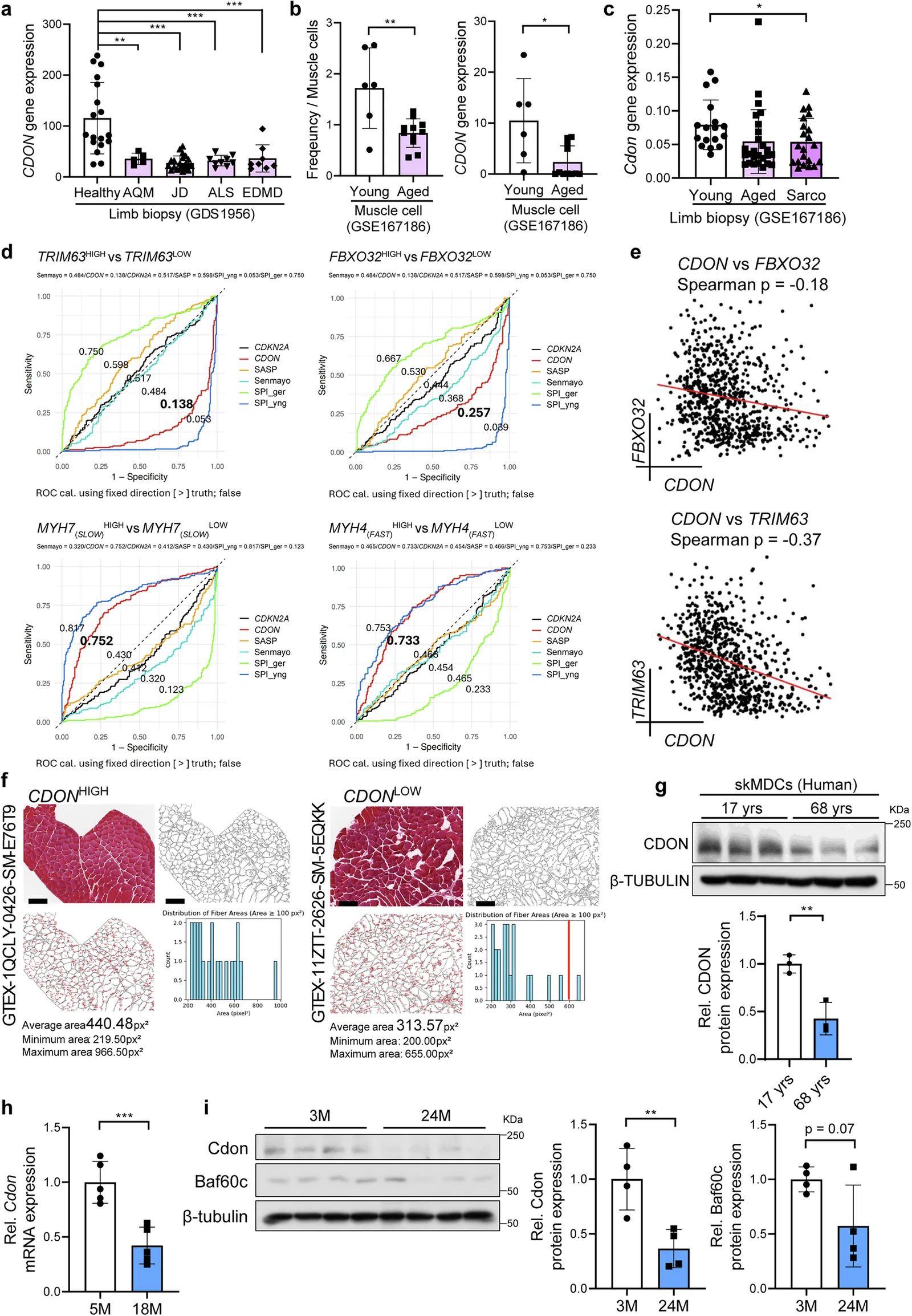
The expression of Cdon in aged muscle and muscle disease. **a** *CDON* gene expression in human limb biopsy samples from a muscle-disease cohort (GDS1956), comparing healthy controls with acute quadriplegic myopathy (AQM), juvenile dermatomyositis (JD), amyotrophic lateral sclerosis (ALS) and Emery–Dreifuss muscular dystrophy (EDMD). **b** Skeletal muscle small nuclear RNA-sequencing dataset (GSE167186) comparing young and aged donors. The left plot shows the frequency of *CDON*-expressing cells within the muscle-cell population, and the right plot shows *CDON* expression levels within muscle cells. **c** Limb biopsy bulk RNA-sequencing data from GSE167186 showing *CDON* expression in young, aged and sarcopenic individuals. **d** Receiver operating characteristic (ROC) curves evaluate *CDON* (primary marker; red) and reference indices in GTEx skeletal muscle, *n* = 891. In the upper panels, *CDON* separates *TRIM63*^HIGH^ vs *TRIM63*^LOW^ (area under the curve, AUC = 0.138) and *FBXO32*^HIGH^ vs *FBXO32*^LOW^ (AUC = 0.257) in an inverse direction. In the lower panels, *CDON* shows higher classification performance for *MYH7*^high^ vs *MYH7*^low^ (AUC = 0.752) and *MYH4*^high^ vs *MYH4*^low^ (AUC = 0.733). Reference curves include *CDKN2A*, SASP, Senmayo, SPI_ger and SPI_yng. **e** Scatter plots show Spearman correlation between *CDON* and the muscle atrophy genes *FBXO32* and *TRIM63* in GTEx skeletal muscle, *n* = 891. Each point represents one sample, and the red line indicates the fitted trend. **f** Quantitative analysis of myofibre cross-sectional area from H&E-stained skeletal muscle sections in a human GTEx-derived cohort. Whole-slide images were acquired at ×20 magnification (0.5 µm/pixel). Fibre areas were measured using a computational pipeline, with pixel-based areas converted to micrometre-scale values using the recorded pixel size; representative examples of *CDON*^HIGH^ and *CDON*^LOW^ samples and the corresponding fibre-area distributions are shown. **g** Immunoblot analysis for *CDON* expression in human skeletal muscle-derived cells (skMDCs). β-tubulin serves as a loading control, *n* = 3. **h** Quantitative real-time PCR analysis for *Cdon* mRNA expression in gastrocnemius muscles from young (5-month-old, 5 M) or aged (18 M) mice. *18* *s rRNA* serves as internal control, *n* = 5. **i** Immunoblot analysis for protein expression in gastrocnemius muscle from young (3 M) or aged (24 M) mice. Right graphs indicate quantification of Cdon or Baf60c expressions, *n* = 4. Data are presented as mean ± SD. Statistical significance was determined using one-way ANOVA. *P < 0.05, **P < 0.01 and ***P < 0.001.

To investigate whether Cdon is implicated in muscle atrophy, we first assessed its expression across human muscle disease datasets representing diverse aetiologies. Transcriptomic analysis of limb muscle biopsies from acute quadriplegic myopathy, juvenile dermatomyositis, amyotrophic lateral sclerosis and Emery–Dreifuss muscular dystrophy demonstrated a consistent reduction in *CDON* expression relative to healthy controls (Fig. 1a).

Single-nucleus RNA sequencing further revealed that *CDON* expression was reduced within aged human myofibres (Fig. 1b), a pattern confirmed by bulk RNA-sequencing datasets from sarcopenic individuals (Fig. 1c). Analysis of the Genotype-Tissue Expression (GTEx) cohort (*n* = 891) showed that low *CDON* expression correlated strongly with elevated expression of atrogenes, including *TRIM63* and *FBXO32*, whereas higher *CDON* levels were associated with preserved contractile signatures such as *MYH4* and *MYH7* (Fig. 1d, e). Muscles exhibiting low *CDON* levels also displayed smaller myofiber cross-sectional areas (Fig. 1f), linking reduced expression to structural decline.

At the cellular level, human skMDCs differentiated into myotubes showed age-dependent suppression of CDON, with cells derived from a 68-year-old donor exhibiting substantially lower CDON protein expression than a 17-year-old donor (Fig. 1g). Similar age-associated reductions in *Cdon* mRNA and protein were observed in skeletal muscle from aged mice (Fig. 1h, i), accompanied by decreased expression of Baf60c, a regulator of myogenic and glycolytic programs.

Together, these findings identify Cdon decline as a conserved feature of human and murine muscle aging and disease and show that low Cdon correlates with atrophy-associated transcriptional programs and diminished contractile identity.

### Meloxicam is identified as a potent Cdon inducer in muscle cells and enhances myogenesis

**Fig. 2:**
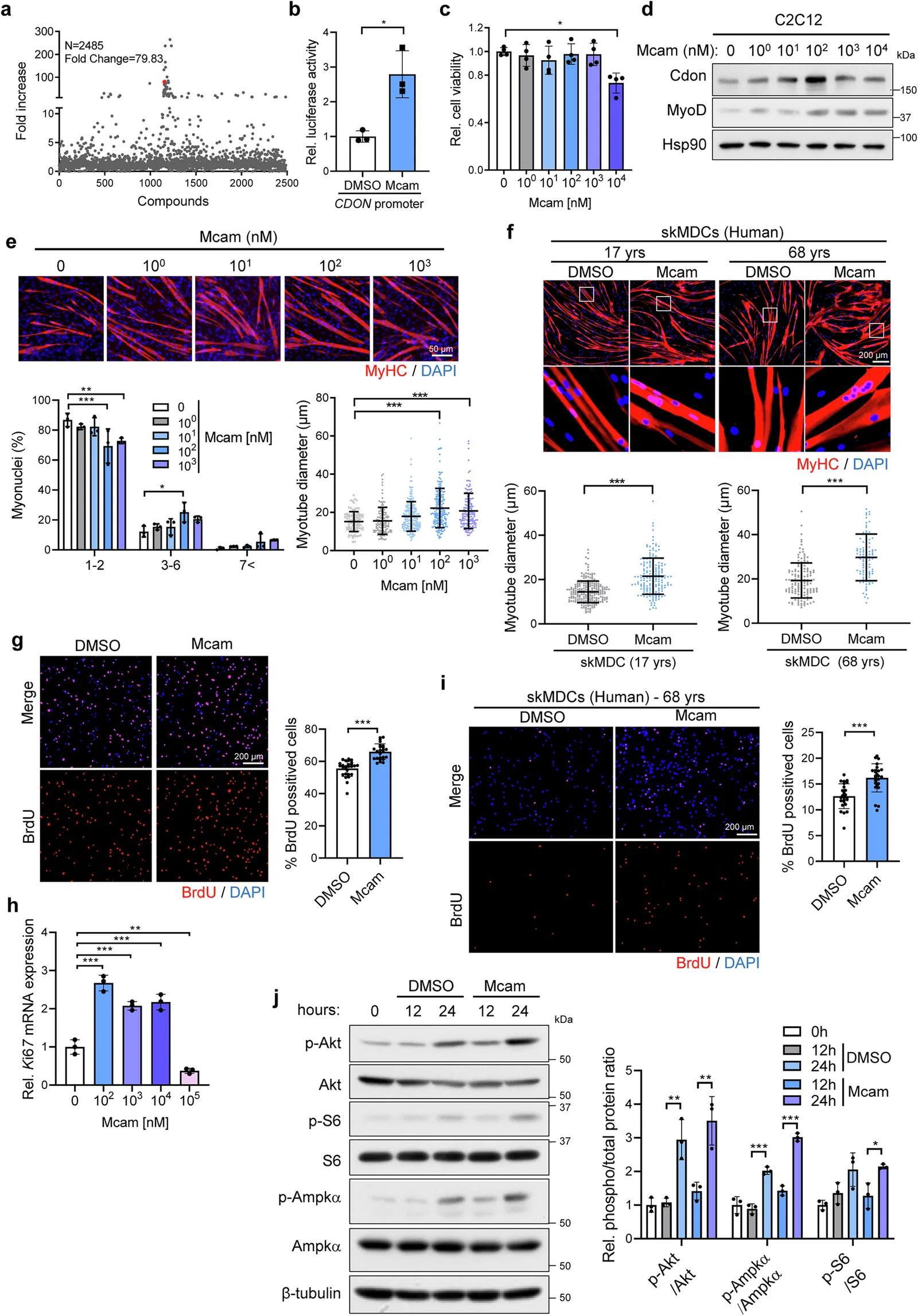
Identification of the Cdon inducer meloxicam and its effects on myogenesis. **a** High-throughput luciferase screening of *Cdon* promoter activity in C2C12 cells treated with 2,485 FDA-approved compounds. The red dot indicates activity of meloxicam (Mcam). **b** Human *CDON* promoter activity in DMSO- or Mcam-treated C2C12 myoblasts, *n* = 3. **c** Cell viability of C2C12 cells treated with Mcam at the indicated concentrations, *n* = 4. **d** Immunoblot analysis for the indicated protein expression in C2C12 cells, *n* = 3. **e** Immunostaining for MyHC (red) in C2C12 cells treated with Mcam at the indicated concentrations (top). Myonuclei were visualized by DAPI (blue). Bottom graphs indicate percentage of nuclei contained within multinucleated myotubes (myonuclei (%), *n* = 3) and myotube diameters (μm), *n* = 97 ~ 183 myotubes. **f** Immunostaining for MyHC (red) in DMSO- or Mcam-treated human skeletal muscle-derived cell (skMDCs), bottom graphs indicate myotube diameters (μm), (17 years; *n* = 175 ~ 220, 68 years; *n* = 100 ~ 140). **g** Immunostaining of BrdU (red) in C2C12 cells. Right graph indicates percentage of BrdU-positive cells (Veh *n* = 27, Mcam = *n* = 25). **h** Quantitative real-time PCR analysis of *Ki67* gene expression in C2C12 cells, *n* = 3. **i** Immunostaining of BrdU (red) in human skMDCs. Right graph indicates percentage of BrdU-positive cells, *n* = 25. **j** Immunoblot analysis for indicated protein expression in C2C12 cells after 12 and 24 h of Mcam treatment. Right graph shows phosphorylation ratio of indicated proteins, *n* = 3. Data are presented as mean ± SD. Statistical significance was determined using unpaired Student’s *t* test (panels **b**, **f**, **g**, **i**) and one-way analysis of variance (panel **a**, **c**, **e**, **h**, **j**). *P < 0.05, **P < 0.01 and ***P < 0.001.

To identify pharmacological activators of Cdon, we performed a Cdon promoter-driven luciferase screen in C2C12 myoblasts. This screening approach identified Mcam as a potent inducer of Cdon reporter activity (Fig. 2a). Treatment with 1 μM Mcam significantly increased reporter activity (Fig. 2b), whereas higher doses (10 μM) reduced cell viability (Fig. 2c), indicating a narrow efficacy window.

Given the established role of Cdon in myogenic progression, we examined the impact of Mcam during C2C12 differentiation. Among the concentrations tested, 100 nM Mcam elicited the strongest induction of Cdon and MyoD protein expression (Fig. 2d), accompanied by increased myotube size and maturation (Fig. 2e). Similar enhancement of myogenic differentiation was observed in human skMDCs derived from 17-year-old and 68-year-old donors (Fig. 2f). At this dose, Mcam also increased proliferation, evidenced by higher Ki67 and BrdU incorporation in C2C12 myoblasts as well as ageing human skMDCs (Fig. 2g–i). Based on this efficacy-to-cytotoxicity ratio, 100 nM Mcam was selected for subsequent experiments.

Mechanistically, Mcam treatment during differentiation increased phosphorylation of Ampk, Akt and S6 within 12–24 h (Fig. 2j), suggesting coordinated activation of metabolic stress signalling and anabolic pathways known to support myogenesis.

These findings demonstrate that Mcam functions as a pharmacological inducer of Cdon and enhances myogenic proliferation and differentiation in murine and human muscle progenitor cells.

### Meloxicam increases muscle mass and function in young mice

**Fig. 3:**
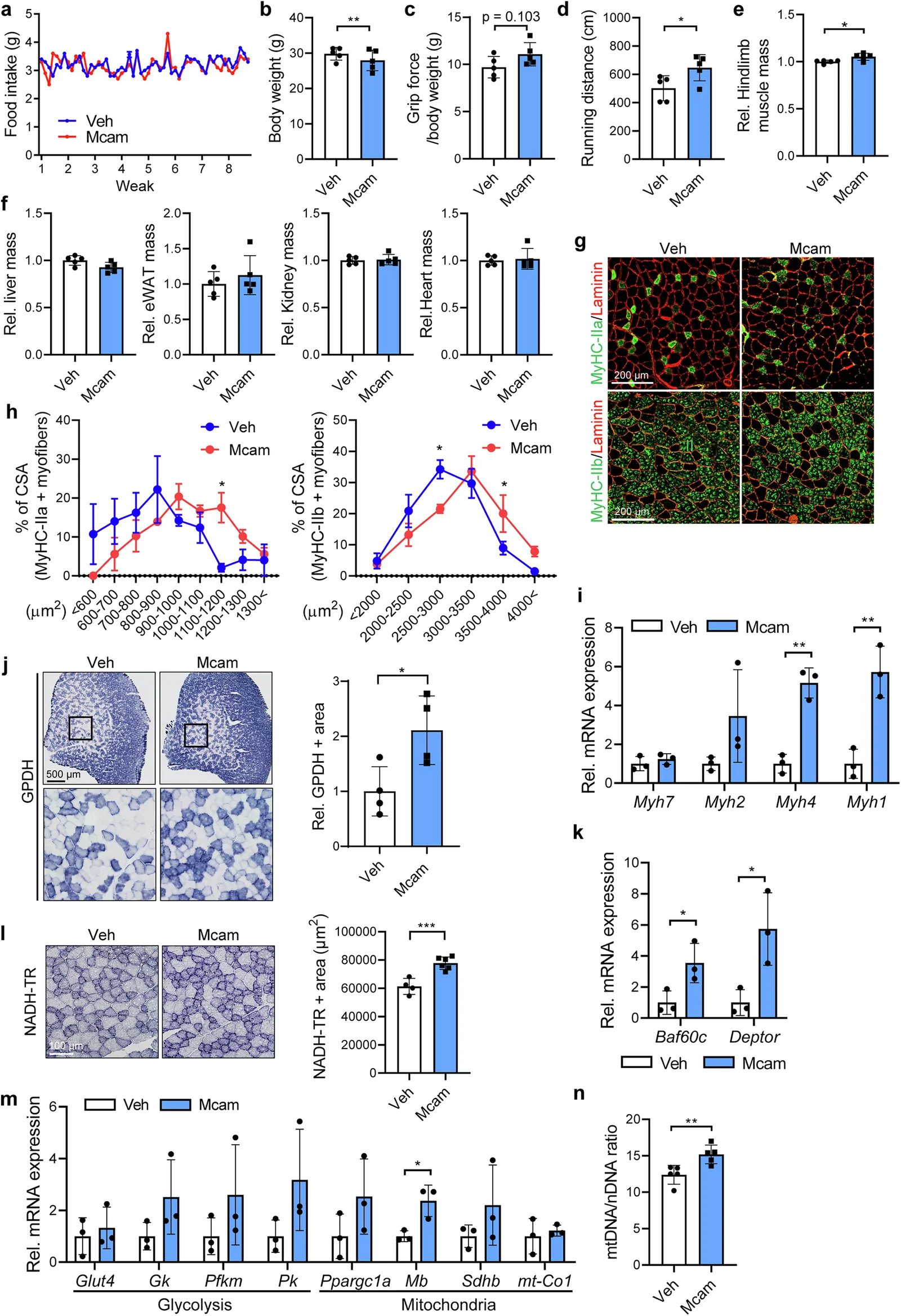
Effects of meloxicam treatment in young mice. Mcam or vehicle (Veh) was orally administered daily to 10-week-old mice for a total of 8 weeks. **a** Food intake (g) was measured at weekly intervals. **b** Body weight (g), *n* = 5. **c** Normalized grip strength (grip force/body weight (g)), *n* = 5. **d** Running distance (cm) measured by treadmill running to exhaustion, *n* = 5. **e** Relative hindlimb muscle mass, calculated as the sum of gastrocnemius, soleus (Sol), tibialis anterior (TA) and extensor digitorum longus muscle mass, normalized to body weight, *n* = 5. **f** Relative mass of liver, epididymal white adipose tissue, kidney and heart, *n* = 5. **g**, Immunostaining for MyHC-Ⅱa (top, green) MyHC-Ⅱb (bottom, green) and laminin (red) in TA muscle. **h** Percentage of cross-sectional area (CSA) positive for MyHC-Ⅱa (right) or MyHC-Ⅱb (left), as determined by immunohistochemical analysis, *n* = 3. **i** Quantitative real-time PCR (qRT-PCR) analysis of *MyHC* isoform messenger RNA (mRNA) expression in TA muscles, *n* = 3. **j** GPDH enzyme activity staining in TA muscles. Right graph indicates relative GPDH staining positive (+) staining area. *n* = 5. **k** qRT-PCR of indicated mRNA expression in TA muscles, *n* = 3. **l** NADH-TR enzyme activity staining in TA muscles. Right graph indicates NADH-TR + staining area (μm^2^) (Veh *n* = 4, Mcam *n* = 6). **m** qRT-PCR analysis of indicated mRNA expression in TA muscles, *n* = 3. **n** qRT-PCR analysis of the mitochondrial DNA (*mt-Co2*) to nuclear DNA (nDNA; *Actb*) ratio in gastrocnemius muscle, *n* = 5. Data are presented as mean ± SD, except for panel **h** which is presented as mean ± SEM. Statistical significance was determined using unpaired Student’s *t* test. *P < 0.05, **P < 0.01 and ***P < 0.001.

To determine whether the myogenic effects of Mcam translate in vivo, 10-week-old mice received daily oral Mcam (0.02 mg/kg) or vehicle for 2 months. Treatment did not alter food intake but resulted in a modest reduction in body weight relative to vehicle controls (Fig. 3a, b). Despite this reduction, Mcam-treated mice exhibited improved muscle function (Fig. 3c, d) and increased hindlimb muscle mass without significant changes in the weights or morphology of non-muscle organs including the heart, liver, kidney or white adipose tissue (Fig. 3e, f; Supplementary Fig. 1a, b).

In TA muscle, Mcam enlarged type IIA and IIB myofibres and increased expression of fast MyHC isoforms, while not affecting type I *Myh7* (Fig. 3g–i). Enzymatic histochemistry revealed enhanced glycolytic (GPDH) and oxidative (NADH-TR) activities (Fig. 3j, k), indicating metabolic remodelling. Gene expression analysis showed increased abundance of glycolytic regulators (*Baf60c* and *Deptor*), glucose-metabolism genes (*Glut4*, *Gk*, *Pfkm* and *Pk*) and mitochondrial regulators (*Ppargc1a*, myoglobin (*Mb*) and *Sdhb*) (Fig. 3l, m), accompanied by elevated mitochondrial DNA content (Fig. 3n).

These findings indicate that Mcam increases muscle mass and enhances endurance-associated metabolic capacity in young sedentary mice by promoting fast fibre growth and coordinated glycolytic and oxidative metabolic remodelling.

### Meloxicam prevents age-related muscle wasting and supports fast fibre integrity

**Fig. 4:**
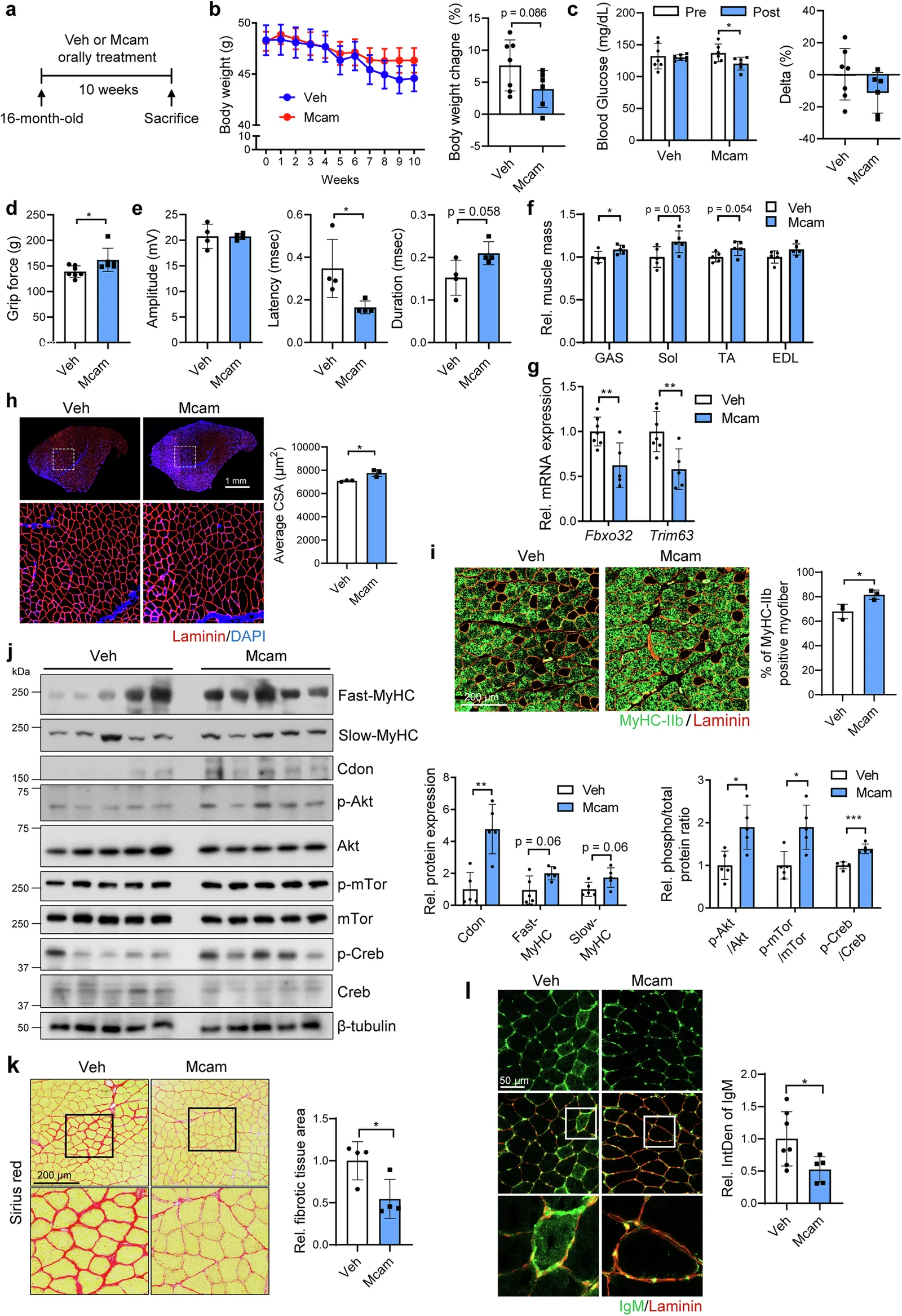
Effects of meloxicam treatment in aged mice. **a** Experimental scheme of aged mice study. Mcam or Veh was orally administered daily to 16-month-old male mice for a total of 10 weeks. **b** Body weight (g) was measured at weekly intervals. Right graph indicates body weight change (%) before and after Mcam or Veh treatment (Veh *n* = 7, Mcam *n* = 6). **c** Blood glucose (mg/dl) was measured before and after Mcam or Veh treatment. Right graph indicates blood glucose change (Delta, %) (Veh *n* = 7, Mcam *n* = 6). **d** Grip force (*g*) (Veh *n* = 7, Mcam *n* = 6). **e** EMG was performed to assess muscle function, measured in terms of amplitude (mV), latency (ms) and duration (ms) (*n* = 4). **f** Relative mass of hindlimb muscles, including gastrocnemius (GAS), soleus (Sol), tibialis anterior (TA) and extensor digitorum longus (EDL), normalized to body weight, *n* = 5. **g** quantitative real-time PCR analysis for muscle atrophy marker-mRNA in GAS muscles (Veh *n* = 7, Mcam *n* = 5). **h** Immunostaining of laminin (red) in TA muscles. Right graph indicates average cross-sectional area (CSA) (μm^2^) (*n* = 3). **i**, Immunostaining MyHC-Ⅱb (green) and laminin (red) in TA muscle. Right graph indicates percentage of MyHC-Ⅱb positive myofibre (*n* = 3). **j** Immunoblot analysis for indicates protein expression in GAS muscles. The right graphs show the expression levels (left) and phosphorylation ratios (right) of the indicated proteins, *n* = 5. **k** Picro Sirius red staining in TA muscles. Right graph indicates relative fibrotic area (red), *n* = 4. **l** Immunostaining for IgM (green) and laminin (red) in TA muscles (Veh *n* = 7, Mcam *n* = 5). Data are presented as mean ± SD. Statistical significance was determined using unpaired Student’s *t* test. *P < 0.05, **P < 0.01 and ***P < 0.001.

We next investigated whether Mcam mitigates age-associated muscle decline. Sixteen-month-old mice received Mcam for 10 weeks (Fig. 4a). Vehicle-treated aged mice exhibited progressive body weight loss, whereas Mcam slowed this decline and modestly reduced blood glucose levels (Fig. 4b, c). Functionally, Mcam improved grip strength and enhanced neuromuscular conduction, demonstrated by shortened compound muscle action potential latency and increased signal duration (Fig. 4d, e).

Mcam treatment increased skeletal muscle mass and reduced gene expression of *Fbxo32* and *Trim63* without significantly affecting other organs (Fig. 4f, g; Supplementary Fig. 2a–c) and did not induce gastrointestinal toxicity (Supplementary Fig. 2d). Cross-sectional area of TA myofibres increased following Mcam treatment, with the most pronounced effects in type IIB fibres, which also showed higher relative abundance (Fig. 4h, i; Supplementary Fig. 2e). These structural improvements were accompanied by increased gene expression levels of type IIB myofibre isoforms (*Myh2*, *Myh4* and *Myh1*) (Supplementary Fig. 2f). At the protein level, Cdon fast-MyHC proteins, and phosphorylation of Akt and mTor was increased by Mcam treatment; these results suggest enhanced anabolic signalling (Fig. 4j). Mcam-treated muscles exhibited declining tissue damage characteristics, including fibrosis and IgM deposition (Fig. 4k, l).

RNA-sequencing revealed that Mcam downregulated slow fibre-associated genes (*Myl2*, *Myl3*, *Myh7* and *Tnnt1*) and upregulated genes linked to extracellular matrix remodelling, membrane-associated signalling and mitochondrial ribosomal proteins (Supplementary Fig. 2g). Gene set variation analysis demonstrated activation of programs associated with cell fate commitment, proliferation, fibre development and tissue regeneration, accompanied by suppression of apoptosis and contractile gene signatures.

Next, to assess whether changes in prostaglandin signalling or prostaglandin E2 (PGE₂) levels, both critical for muscle homeostasis, occurred, we measured *Ptgs2* expression and PGE₂ levels in muscle and plasma^29,30^. *Ptgs2* mRNA in GAS muscles showed a trend towards an increase in aged mice compared with young mice (*P* = 0.09). In plasma, PGE₂ levels were elevated in aged mice and were significantly reduced by Mcam treatment, indicating a systemic effect while preserving physiological prostaglandin signalling in muscle (Supplementary Fig. 3a–c). At the dose used (0.02 mg/kg) in this study, Mcam did not significantly alter muscle PGE₂ levels, supporting the notion that the protective effects of Mcam against age-related sarcopenia are unlikely to be due to sustained COX-2 inhibition.

Together, these findings indicate that Mcam attenuates age-related muscle atrophy by preserving fast-fibre structure, promoting anabolic signalling and improving neuromuscular function.

### Meloxicam enhances mitochondrial metabolism and quality control in aged muscle and liver

**Fig. 5:**
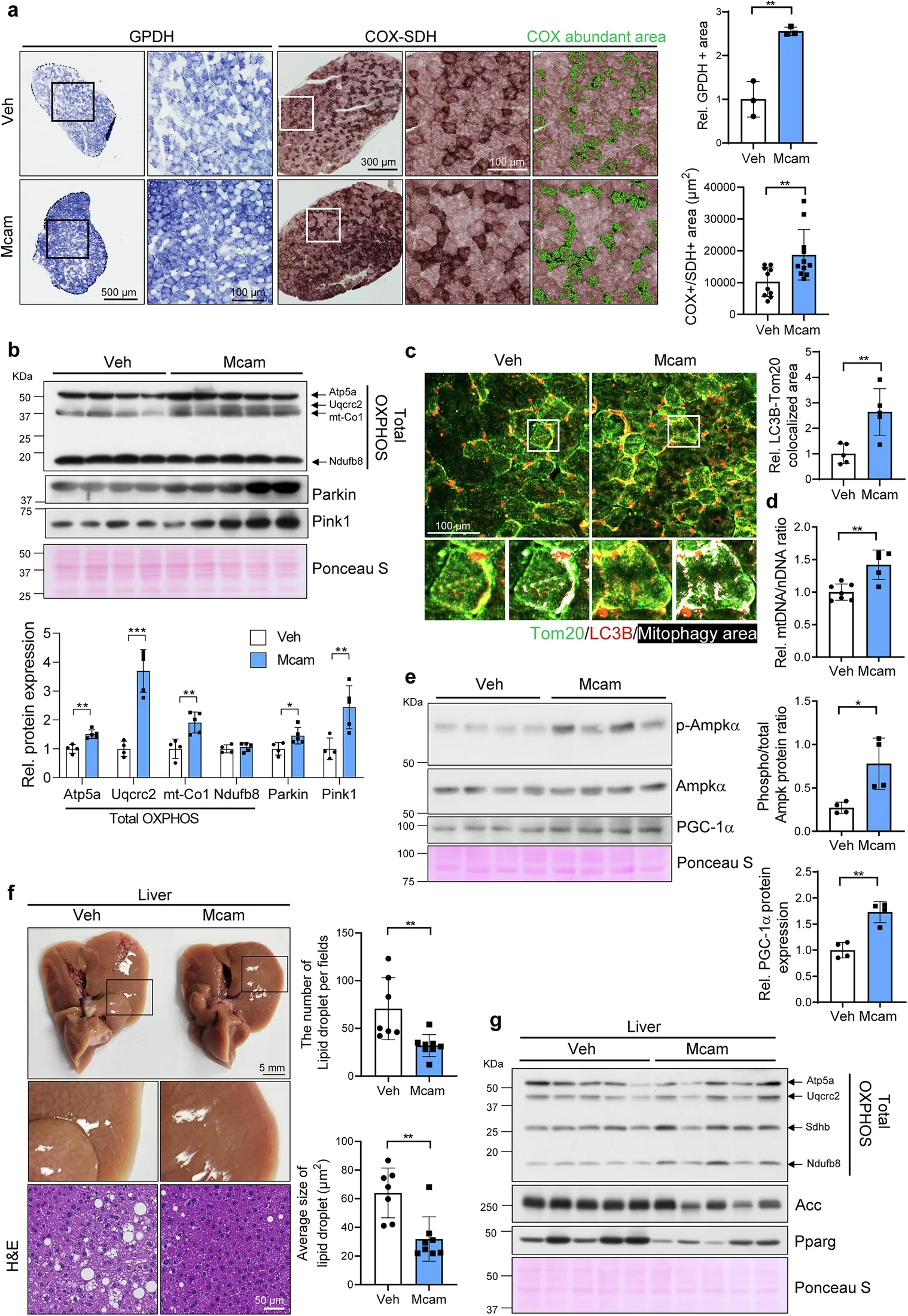
Effects of meloxicam on liver and muscle metabolic capacity in aged mice. **a** GPDH and COX-SDH enzyme activity staining in extensor digitorum longus muscles. Top right graph indicates relative GPDH staining-positive area (*n* = 3). Bottom right graph indicates COX/SDH-positive staining area (μm^2^) (Veh *n* = 10, Mcam *n* = 11). **b** Immunoblot analysis for indicated protein expression in GAS muscles. The bottom graph shows relative expression levels of indicated proteins (Veh *n* = 4, Mcam *n* = 5). **c** Immunostaining for LC3B (red) and Tom20 (green) in TA muscles. Right graph indicates relative LC3B-Tom20 colocalized area (*n* = 5). **d** Quantitative real-time PCR analysis for ratio of mtDNA/nDNA in GAS muscles (Veh *n* = 7, Mcam *n* = 6). **e** Immunoblot analysis for indicated protein expression in quadriceps muscles. Top right graph indicates phosphorylated Ampkα levels. Bottom right graph indicates relative protein expression levels of PGC-1α (*n* = 4). **f** The gross images of liver (top) and haematoxylin and eosin (H&E) staining (bottom) in Veh- or Mcam-treated mice. Top right graph indicates the number of lipid droplets in liver. Bottom right graph indicates average size (μm^2^) of lipid droplets (Veh *n* = 7, Mcam *n* = 8). **g** Immunoblot analysis for indicated protein expression in liver (*n* = 5). Data are presented as mean ± SD. Statistical significance was determined using unpaired Student’s *t* test. *P < 0.05, **P < 0.01 and ***P < 0.001.

Because mitochondrial dysfunction is a hallmark of muscle ageing, we examined whether Mcam influences mitochondrial metabolism in aged mice. Histochemical staining revealed that Mcam-treated muscle contained a higher proportion of strongly GPDH-positive fibre and fibres with enhanced oxidative capacity (COX/SDH-positive) (Fig. 5a). These structural changes were accompanied by increased expression of OXPHOS proteins and elevated levels of the mitophagy regulators Parkin and Pink1 (Fig. 5b).

Consistent with improved mitochondrial clearance, Mcam increased colocalization of the mitochondrial marker Tom20 with the autophagosomal marker LC3B (Fig. 5c), suggesting enhanced mitochondrial quality control. These changes corresponded with increased mitochondrial content and elevated phosphorylation of Ampkα, together with increased expression of PGC-1α (Fig. 5d, e).

Given that skeletal muscle and liver are two major metabolic organs that coordinate systemic metabolism, liver tissue was additionally examined. Mcam treatment markedly reduced hepatic lipid accumulation (Fig. 5f) and enhanced hepatic OXPHOS protein expression, and suppressed lipogenic regulators acetyl-CoA carboxylase (Acc) and peroxisome proliferator-activated receptor gamma (Pparγ) (Fig. 5g; Supplementary Fig. 4a, b).

Together, these findings demonstrate that Mcam promotes mitochondrial biogenesis and enhances mitochondrial quality control in aged muscle and liver, supporting coordinated improvements in tissue redox and metabolic homeostasis.

### Meloxicam normalizes metabolic dysfunction and cysteine accumulation in aged mice

**Fig. 6:**
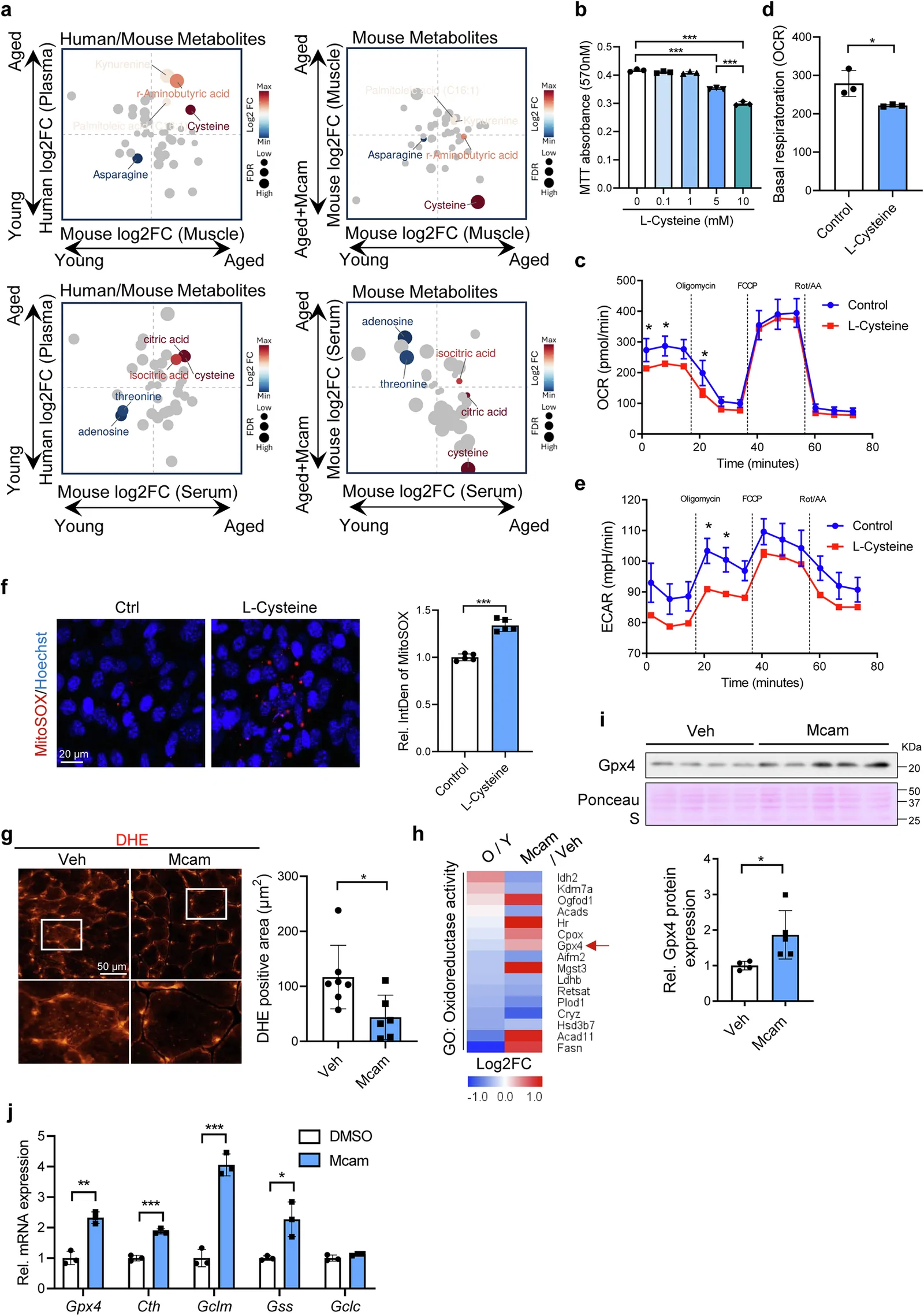
Effects of meloxicam on metabolite profile changes and oxidative stress resistance. **a** Human and mouse metabolite changes during aging and after Mcam treatment were compared using published human plasma metabolomics data and in-house mouse quadriceps muscle and plasma/serum profiling. Scatter plots show cross-species concordance of age-associated metabolite changes and their attenuation by Mcam treatment in aged mice, in both muscle and circulation. Each dot represents a metabolite; colour indicates log2 fold-change (FC), dot size reflects false discovery rate (FDR), and selected metabolites are annotated. **b** MTT absorbance (570 nm) in C2C12 myoblasts treated with the indicated concentrations of L-cysteine (*n* = 3). **c** Oxygen consumption rate (OCR) measurement after treating the indicated drug using seahorse analyser in control- or L-cysteine-treated C2C12 cells (*n* = 3). **d** Basal respiration (OCR) levels in C2C12 cells (*n* = 3). **e** ECAR measurement after treating indicated drug using seahorse analyser in control- or L-cysteine-treated C2C12 cells (*n* = 3). **f** MitoSOX (red) staining in C2C12 cells. Myonuclei were visualized by Hoechst 33342 (blue). Right graph indicates IntDen of MitoSOX (*n* = 5). **g** DHE (red) staining in EDL muscles. Right graph indicates IntDen of DHE (Veh *n* = 7, Mcam *n* = 6). **h** Heatmap showing expression of genes in the oxidoreductase activity gene set in old (O) versus young (Y) muscle (left, GSE145480, old (28 M) *n* = 9, young (8 M) *n* = 8) and in Mcam- versus Veh-treated gastrocnemius muscle (right, *n* = 3). **i** Immunoblot analysis for Gpx4 protein expression in gastrocnemius muscle. The bottom graph indicates relative Gpx4 protein expression (Veh *n* = 4, Mcam *n* = 5). **j** Quantitative real-time PCR analysis for indicated mRNA expression in DMSO- or Mcam-treated C2C12 cells (*n* = 3). Data are presented as mean ± SD. Statistical significance was determined using one-way analysis of variance (panel **b**), unpaired Student’s *t* test (panels **d**, **f**, **g**, **i**, **j**) and two-way analysis of variance (panels **c**, **e**). *P < 0.05, **P < 0.01 and ***P < 0.001.

To examine the metabolic consequences of Mcam treatment, we performed metabolomic profiling of muscle and serum from young and aged mice and compared these findings with plasma metabolomic data from young and sarcopenic individuals^31^ (Fig. 6a). Ageing in mice was associated with elevated γ-aminobutyric acid and cysteine and reduced asparagine in muscle, as well as increased cysteine and tricarboxylic acid (TCA) cycle intermediates in serum. Sarcopenic patients similarly exhibited increased plasma cysteine. Mcam reversed key features of this metabolic profile, reducing γ-aminobutyric acid and cysteine levels in aged muscle and lowering citric acid and cysteine in aged serum, suggesting correction of age-related dysregulation of amino acid and TCA cycle metabolism.

Because aberrant cysteine accumulation resulting from disturbed cysteine metabolism has been linked to redox imbalance and mitochondrial impairment, we examined its functional consequence in vitro^32^. In MTT assays, exposure to elevated concentrations of L-cysteine (5–10 mM) significantly reduced cell viability (Fig. 6b). Moreover, treatment of muscle cells with 1 mM L-cysteine for 24 h markedly suppressed basal mitochondrial respiration and glycolytic activity (Fig. 6c–e), accompanied by increased oxidative stress (Fig. 6f). In agreement with these observations, Mcam-treated aged muscles exhibited reduced oxidative stress, restoration of redox-related transcriptional programs and increased Gpx4 expression at transcript and protein levels (Fig. 6g–i). Mcam treatment also induced redox-related genes including *Gpx4*, *Cth*, *Gclm* and *Gss* in muscle cells (Fig. 6j).

These findings define a cysteine-associated redox axis in muscle ageing and show that Mcam restores metabolic and redox balance in aged muscle by normalizing cysteine levels and enhancing antioxidant defence.

### Meloxicam maintains Cdon under oxidative stress through Ampk-dependent antioxidant and mitochondrial protection

**Fig. 7:**
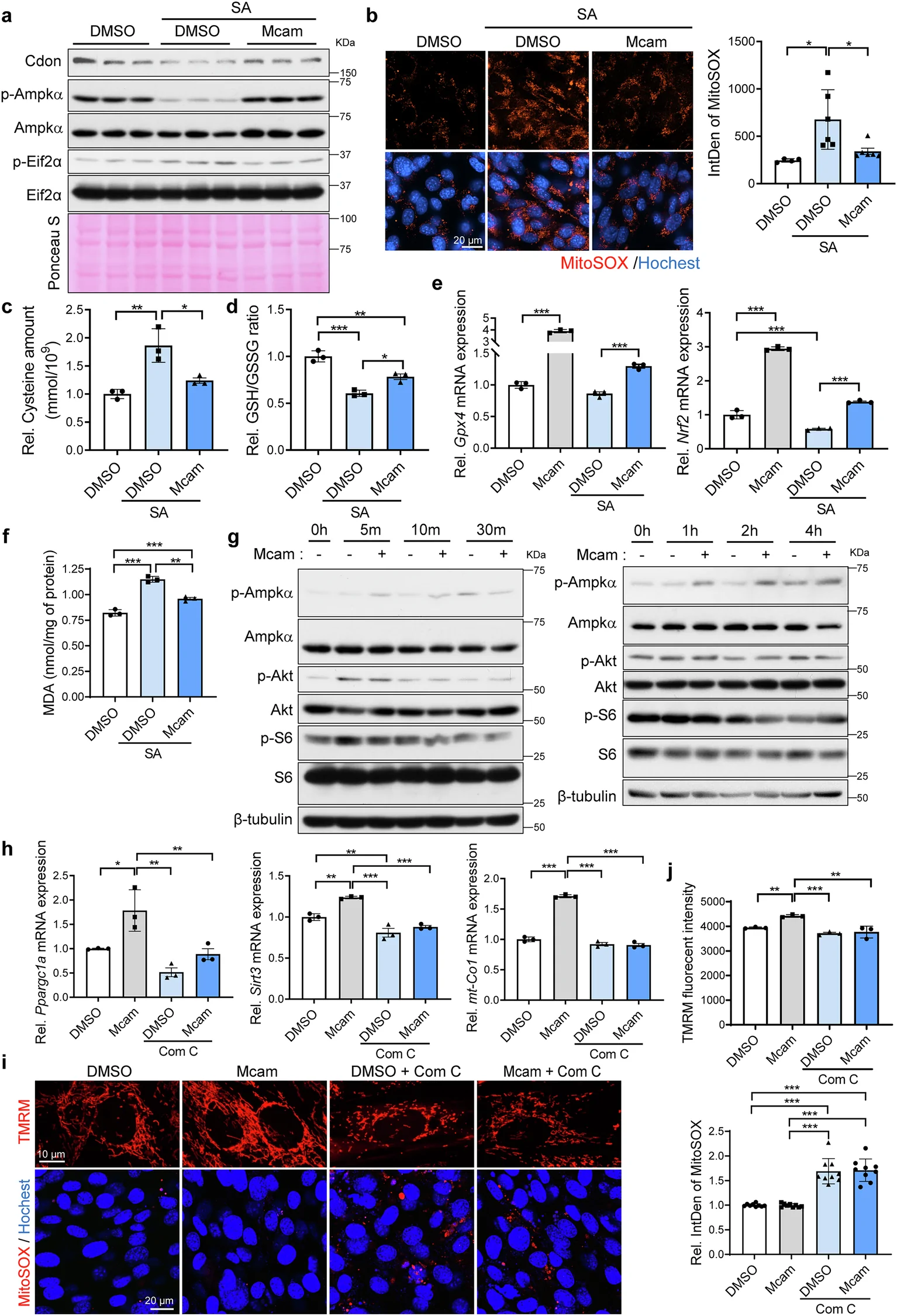
Ampk phosphorylation induced by Mcam treatment. **a** Immunoblot analysis for indicated protein expression in C2C12 cells. Cells were treated with DMSO, sodium arsenate (SA) in combination with DMSO, or SA in combination with Mcam for 24 h (*n* = 3). **b** MitoSOX (red) staining in C2C12 cells. Right graph indicates IntDen of MitoSOX (DMSO *n* = 4, DMSO + SA *n* = 6, Mcam + SA *n* = 6). **c** Relative cysteine amount (mmol/10^9^) in C2C12 cells (*n* = 3). **d** Relative GSH/GSSG ratio in C2C12 cells (*n* = 3). **e** Quantitative real-time PCR (qRT-PCR) analysis for mRNA expression of *Gpx4* (left) and *Nrf2* (right) in C2C12 cells (*n* = 3). **f** MDA concentration (nmol/mg of protein) in C2C12 cells (*n* = 3). **g** Immunoblot analysis for indicated protein expression in C2C12 cells. Cells were starved in DMEM containing 2% HS for 24 h, followed by treatment with DMSO or Mcam, and cell signalling was analysed within 1 h (left) or 4 h (right). **h** qRT-PCR analysis for indicated mRNA expression in C2C12 cells (*n* = 3). **i** TMRM (top, red) or MitoSOX (bottom, red) staining in C2C12 cells. **j** Top graph indicates TMRM fluorescence intensity per well (*n* = 3). Bottom graph indicates relative IntDen of MitoSOX (*n* = 9). Data are presented as mean ± SD. Statistical significance was determined using one-way analysis of variance. *P < 0.05, **P < 0.01 and ***P < 0.001.

To determine how Mcam preserves Cdon expression under oxidative stress, we treated C2C12 cells with SA, a potent inducer of oxidative and translational stress. SA treatment markedly reduced Cdon protein abundance, attenuated Ampkα phosphorylation and increased phosphorylation of Eif2a, consistent with stress-induced translational inhibition (Fig. 7a; Supplementary Fig. 5a). Notably, Mcam restored Cdon expression under these oxidative conditions, concomitantly reactivating Ampk signalling and normalizing p-Eif2a levels. In parallel, Mcam markedly suppressed SA-induced mitochondrial oxidative stress (Fig. 7b), prevented cysteine accumulation (Fig. 7c) and restored the GSH/GSSG ratio (Fig. 7d), collectively indicating enhanced antioxidant capacity and redox homeostasis.

At the transcriptional level, Mcam increased the expression of the antioxidant regulators *Gpx4* and *Nrf2* under both basal and oxidative stress conditions (Fig. 7e), consistent with enhanced cellular redox buffering. Correspondingly, under SA-induced oxidative stress, Mcam reduced levels of the lipid peroxidation marker, malondialdehyde (MDA) (Fig. 7f), in line with the increased *Gpx4* expression. It also upregulated *Sod2*, *Pink1* and *Nrf1* (Supplementary Fig. 5b), implicating improved redox regulation and mitochondrial quality control. Functionally, Mcam significantly enhanced basal mitochondrial respiration under oxidative stress (Supplementary Fig. 5c). Mcam rapidly activated Ampkα phosphorylation within 5 min that was sustained up to 4 h, whereas vehicle treatment produced only a modest and transient response and Mcam did not alter Akt signalling (Fig. 7g).

To determine whether Ampk activity is required for Mcam-mediated effects, we pharmacologically inhibited Ampk signalling using Com C. Ampk inhibition reduced basal Cdon expression, increased mitochondrial ROS production and elevated cysteine levels (Supplementary Fig. 5d–f), phenocopying oxidative stress-associated metabolic imbalance. Importantly, Com C substantially attenuated Mcam-induced Cdon elevation (Supplementary Fig. 5g) and suppressed Mcam-mediated induction of *Ppargc1a,* *Sirt3* and *mt-Co1* (Fig. 7h). Consistent with these findings, Mcam-enhanced mitochondrial function and suppression of oxidative stress were abolished by Ampk inhibition (Fig. 7i, j).

Collectively, these findings demonstrate that Mcam activates Ampk signalling to preserve Cdon expression in the context of oxidative stress, restrain cysteine accumulation and maintain mitochondrial homeostasis. These results support an Ampk-dependent mechanism underlying the protective effects of Mcam against redox-mediated cellular dysfunction.

## Discussion

Age-related skeletal muscle decline arises from the convergence of metabolic impairment, redox imbalance, impaired proteostasis and neuromuscular deterioration^3–5,33^. Although these features are well described, the upstream regulators that coordinate metabolic resilience and structural maintenance remained incompletely defined. In this study, we identify Cdon as a previously unappreciated determinant of adult muscle homeostasis whose decline is conserved across human ageing, sarcopenia, and muscle-wasting disease. Reduced Cdon expression was associated with transcriptional signatures of atrophy, suppression of contractile gene programs, and reduced myofibre size, supporting a contributory role for Cdon loss in the progressive deterioration of ageing muscle.

A major finding of this work is that Mcam restores Cdon expression and reactivates a coordinated program supporting myogenic integrity, metabolic fitness and neuromuscular function. In myogenic cells, Mcam increased Cdon levels, promoted differentiation and enhanced myotube growth. In vivo, Mcam increased muscle mass and function in young mice and mitigated age-related muscle loss, accompanied by improvements in mitochondrial performance and neuromuscular conduction. These observations are consistent with prior studies demonstrating that Cdon is required for satellite cell maintenance and neuromuscular junction stability, with Cdon deficiency leading to impaired regeneration and synaptic fragmentation^19,20^. Together, these findings indicate that restoration of Cdon influences multiple components of muscle homeostasis.

Our data further implicate dysregulated cysteine metabolism and oxidative stress as upstream contributors to Cdon decline. Ageing was associated with cysteine accumulation in mouse serum and muscle, as well as in plasma from individuals with sarcopenia. Experimentally increasing cysteine impaired mitochondrial respiration and increased oxidative stress in muscle cells, consistent with previous reports linking sulphur amino acid imbalance to mitochondrial dysfunction and ferroptosis susceptibility^31,34,35^. Mcam corrected cysteine accumulation, restored GSH/GSSG ratio and increased Gpx4 expression, suggesting amelioration of thiol-redox stress. These findings support a model in which cysteine-driven oxidative imbalance suppresses Cdon expression, and Mcam acts upstream to restore redox homeostasis and preserve Cdon under stress conditions.

The metabolic and redox disturbances observed in aged muscle, including altered cysteine metabolism and disrupted glutathione buffering, are consistent with established models positioning oxidative stress and thiol-redox dysregulation as central contributors to skeletal muscle ageing^36–39^. In this context, the increase in Gpx4 expression and the restoration of the GSH/GSSG ratio following Mcam treatment are particularly notable. Gpx4 is a key regulator of lipid peroxide detoxification and plays a critical role in suppressing ferroptotic damage^40^. Thus, the normalization of Gpx4 and glutathione homeostasis suggests that Mcam alleviates oxidative stress and protects mitochondrial integrity in aged muscle. Together with the observed requirement for Ampk activity, these findings support a model in which Mcam restores redox balance and mitochondrial function by stabilizing a Ampk–Cdon regulatory axis under conditions of oxidative stress.

Ampk emerged as a central mediator of these protective effects. Mcam rapidly activated Ampk and pharmacological inhibition abolished Mcam-induced Cdon expression, suppressed antioxidant gene induction and prevented improvements in mitochondrial respiration. These findings establish Ampk as a key signalling node linking Mcam treatment with enhanced redox defences and mitochondrial remodelling. Notably, Ampk activation occurred alongside controlled Akt-mTOR signalling, resembling the integrated metabolic–anabolic environment required for effective myogenesis and muscle maintenance in contrast to pathological mTORC1 hyperactivation in ageing muscle^41–43^.

Although Ampk and Akt-mTOR signalling are traditionally viewed as opposing pathways promoting catabolic and anabolic processes respectively, accumulating evidence indicates that they can be co-activated in skeletal muscle under specific physiological conditions. During exercise and myogenic differentiation, transient Ampk activation promotes metabolic adaptation, mitochondrial remodelling and redox homeostasis whereas Akt-mTOR signaling drives protein synthesis and myofibre growth^41,44^. Consistently, Mcam induced concurrent activation of Ampk and mTOR signalling in differentiating C2C12 cells (Fig. 2j) and aged muscle (Figs. 4j, 5e) and was associated with enhanced myogenic differentiation and CREB-dependent transcription.

Importantly, the functional outcome of Ampk activation depends on its temporal dynamics and signalling context. Chronic low-level activation is typically associated with catabolic responses whereas transient or robust activation promotes metabolic adaptation, mitochondrial biogenesis and beneficial anabolic responses^45–49^. In this study, Mcam-induced Ampk activation did not trigger excessive catabolism and muscle mass was preserved or modestly increased, suggesting a physiological, exercise-mimetic activation pattern (Fig. 4f).

The increase in mTOR signalling is likely to be linked to Cdon upregulation. Although direct activation of mTOR by Cdon has not been reported, Cdon enhances Akt-mTOR signalling via interactions with Pkn2 and Fgfr1 and supports anabolic processes during myogenesis^19,50^. Our findings suggest that Mcam promotes skeletal muscle homeostasis by coordinating metabolic and anabolic signalling with Ampk-dependent regulation of redox balance and mitochondrial function operating alongside Cdon-mediated Akt-mTOR activation.

Despite these insights, several questions remain. The temporal dynamics, subcellular localization and enzymatic activity of Ampk during chronic Mcam treatment are unclear. Addressing these will be important for understanding long-term muscle adaptation and refining therapeutic strategies targeting this pathway.

The improvements in neuromuscular function observed in aged mice treated with Mcam are consistent with previous findings implicating Cdon in neuromuscular junction integrity and motor neuron health. The extent to which Mcam directly influences neural components versus acting primarily through muscle remains to be determined. Beyond skeletal muscle, the reduction of hepatic lipid accumulation and enhancement of liver OXPHOS proteins suggest broader metabolic benefits that may arise from improved muscle–liver communication or direct hepatic actions.

This study has limitations. The molecular target through which Mcam activates Ampk remains to be elucidated. The cell-type-specific roles of Cdon in satellite cells, mature myofibres, and motor neurons require further investigation, as do the long-term effects and safety of chronic Mcam treatment. Finally, whether the Ampk–Cdon axis is similarly restored in aged human muscle will require clinical evaluation.

In summary, this study identifies Cdon as a key regulator of muscle maintenance and demonstrates that its age-related decline contributes to metabolic, redox and neuromuscular dysfunction. Pharmacological induction of Cdon through Mcam reestablishes an Ampk-dependent redox–metabolic axis that supports mitochondrial function, reduces oxidative stress, enhances neuromuscular conduction and preserves muscle mass and strength. These findings position the Ampk-Cdon pathway as a potential therapeutic entry point for sarcopenia and muscle degeneration and provide a framework for further investigation into small-molecule strategies targeting redox and metabolic resilience in ageing skeletal muscle.

## Supplementary information

## Data Availability

The data that support the findings of this study are available from the corresponding author. J.S.K. upon reasonable request. **Supplementary information** accompanies the manuscript on the Experimental & Molecular Medicine website (http://www.nature.com/emm/).
