# Supplementary material for: Meloxicam protects against muscle and metabolic decline in ageing through Cdon restoration and oxidative stress modulation
Source: Exp Mol Med. 2026 Aug 12;58(8):2746–61. doi: 10.1038/s12276-026-01804-1 (PMC13538359; doi:10.1038/s12276-026-01804-1)
Supplement: Supplementary file 1 [file 12276_2026_1804_MOESM1_ESM.pdf]

Supplementary Materials for

Meloxicam Protects Against Muscle and Metabolic Decline in Aging Through Cdon Restoration and Oxidative Stress Modulation

Ju-Hyeon Bae, Hyun-Kyung So, Yideul Jeong, Jeongmin Park, June Kim, Tae Young Kim, Young-Eun Leem, Tuan Anh Vuong, Jiwoong Jang, Moongi Ji, Hyon-Seung Yi, Il-Young Kim, Man-Jeong Paik, Sang-Jin Lee, Gyu-Un Bae*, Jong-Sun Kang*

This file includes:

Supplementary Figures 1 to 5

Supplementary Tables 1 to 2

Supplementary Figures

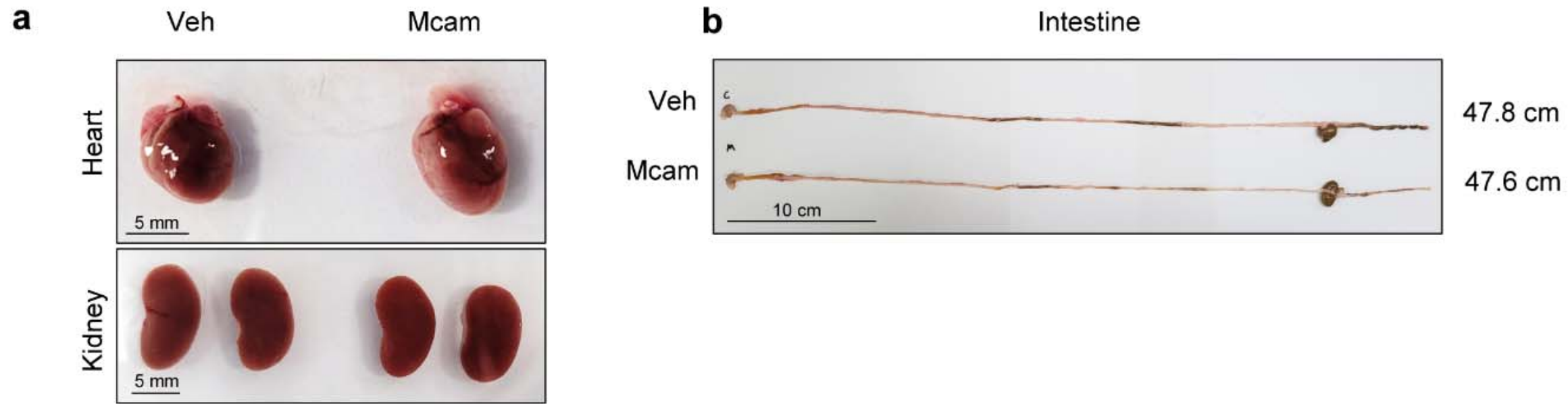

Supplementary Fig. 1: Gross images of organs after Mcam administration in young mice. **a** Representative gross image of heart (upper) or kidney (lower) from Veh- or Mcam treated young mice. **b** Representative gross image of intestine from Veh- or Mcam treated young mice.

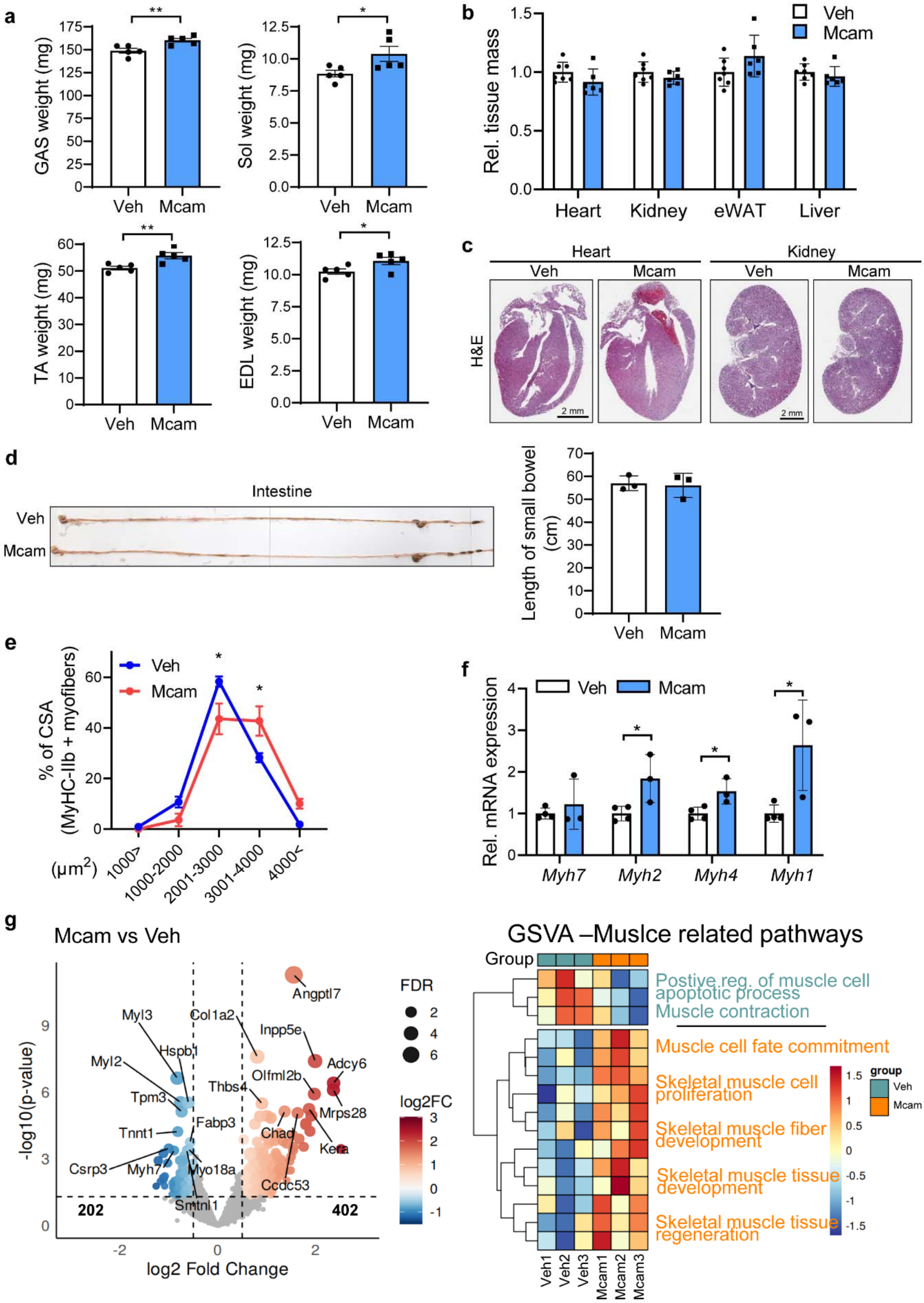

a
GAS weight (mg)
Sol weight (mg)
TA weight (mg)
EDL weight (mg)
Veh
Mcam
b
Rel. tissue mass
Heart
Kidney
eWAT
Liver
c
Heart
Kidney
H&E
2 mm
d
Intestine
Length of small bowel (cm)
e
% of CSA (MyHC-IIb + myofibers)
(μm²)
1000>
1000-2000
2001-3000
3001-4000
4000<
f
Rel. mRNA expression
Myh7
Myh2
Myh4
Myh1
g
Mcam vs Veh
-log10(p-value)
log2 Fold Change
FDR
log2FC
202
402
Angptl7
Myl3
Col1a2
Inpp5e
Hspb1
Myl2
Adcy6
Thbs4
Olfml2b
Tpm3
Mrps28
Fabp3
Tnnt1
Chad
Kera
Csrp3
Myh7
Myo18a
Ccdc53
Smtnl1
GSVA –Muslce related pathways
Group
Postive reg. of muscle cell apoptotic process
Muscle contraction
Muscle cell fate commitment
Skeletal muscle cell proliferation
Skeletal muscle fiber development
Skeletal muscle tissue development
Skeletal muscle tissue regeneration
group
Veh1
Veh2
Veh3
Mcam1
Mcam2
Mcam3

Supplementary Fig. 2: Effects of Mcam treatment in aged mice. a Absolute mass of hindlimb muscles including GAS, Sol, TA and EDL muscles in Veh- or Mcam treated aged mice, $n = 5$. **b** Relative organ or tissue weight from Veh- or Mcam treated aged mice (Veh; $n = 7$, Mcam; $n = 6$). **c** Representative H&E staining image of heart (Left) or kidney (Right) in Veh- or Mcam treated aged mice. **d** Representative gross image of intestine in Veh- or Mcam treated aged mice. Right graph indicates length of small bowel (cm), $n = 3$. **e** Percentage of myofiber CSA positive for MyHC-IIb in Fig. 4i, $n = 3$. **f** qRT-PCR analysis for *MyHC* isoform genes in GAS muscles in Veh- or Mcam treated aged mice, (Veh; $n = 4$, Mcam; $n = 3$). **g** Transcriptomic profiling of aged mice was performed to assess gene-expression changes induced by Mcam treatment relative to vehicle controls, and pathway-level activity was summarized using gene set variation analysis (GSVA) focused on muscle-related programs, $n = 3$. (Left) A volcano plot displays differential expression between Mcam-treated and vehicle-treated aged mice, with the x-axis showing log2 FC (Mcam vs Veh) and the y-axis showing −log10(p-value). (Right): A heatmap shows GSVA scores for muscle-related pathways across the vehicle and Mcam groups, with hierarchical clustering and a color scale indicating relative pathway activity. Data are presented as mean ± ± SEM (panel a, e) or SD (panel b, d and f). Statistical significance was determined using unpaired Student's t-test. $^*p < 0.05$, $^{**}p < 0.01$, and $^{***}p < 0.001$

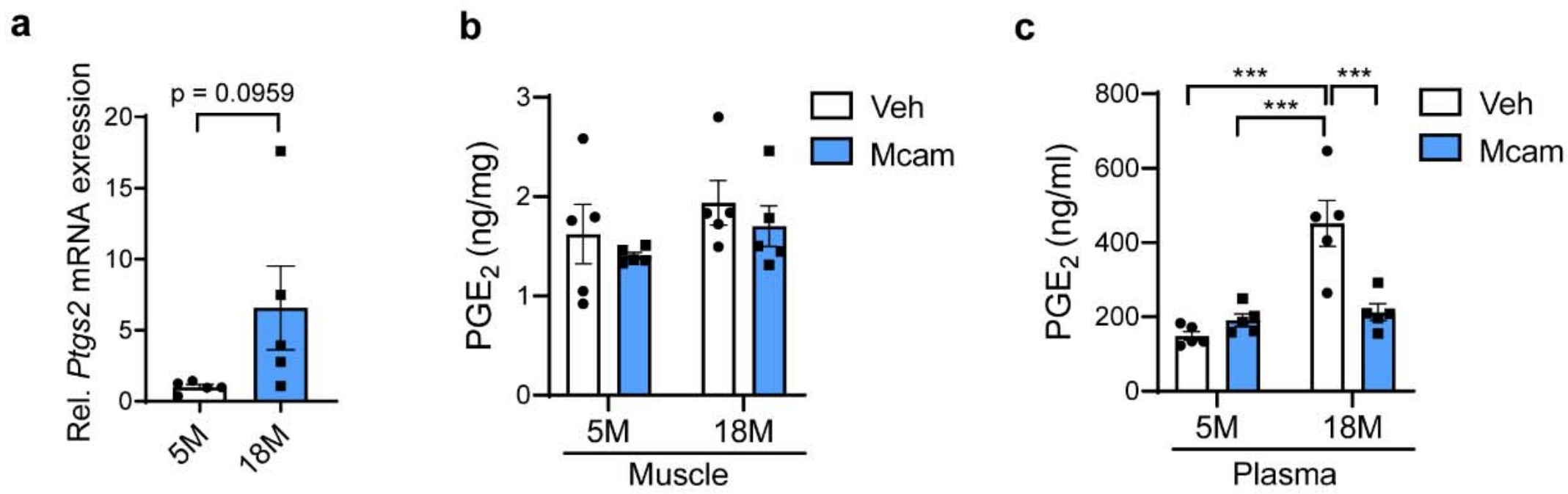

Supplementary Fig. 3: PGE_2 levels in Veh or Mcam treated mice. a Relative *Ptgs2* mRNA expression in GAS muscles from young (5M) or aged (18M) mice, $n = 5$. **b** PGE_2 (ng/mg tissue) levels in GAS muscles from Veh- or Mcam treated mice of indicated age, $n = 5$. **c** PGE_2 (ng/ml plasma) levels in plasma from Veh- or Mcam treated mice of indicated age, $n = 5$. Data are presented as mean ± SEM. Statistical significance was determined using unpaired Student's t-test (panel a) and two-way ANOVA (panel b and c). $*p < 0.05$, $**p < 0.01$, and $***p < 0.001$

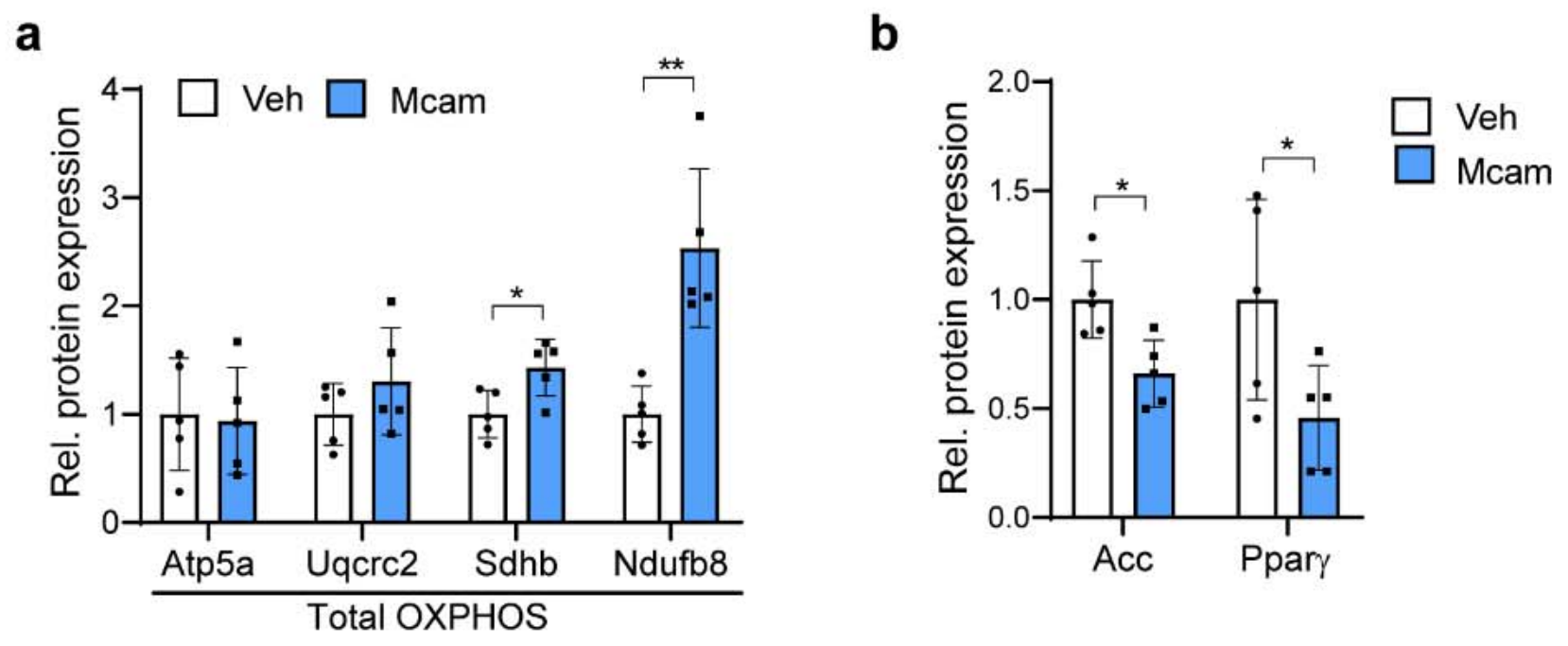

Supplementary Fig. 4: Effects of Mcam in liver from aged mice. a Relative total OXPHOS protein expression in Fig. 5g, *n* = 5. **b** Relative Acc and Pparγ protein expression in Fig. 5g, *n* = 5. Data are presented as mean ± SD. Statistical significance was determined using unpaired Student's t-test. *$p < 0.05$, **$p < 0.01$, and ***$p < 0.001$

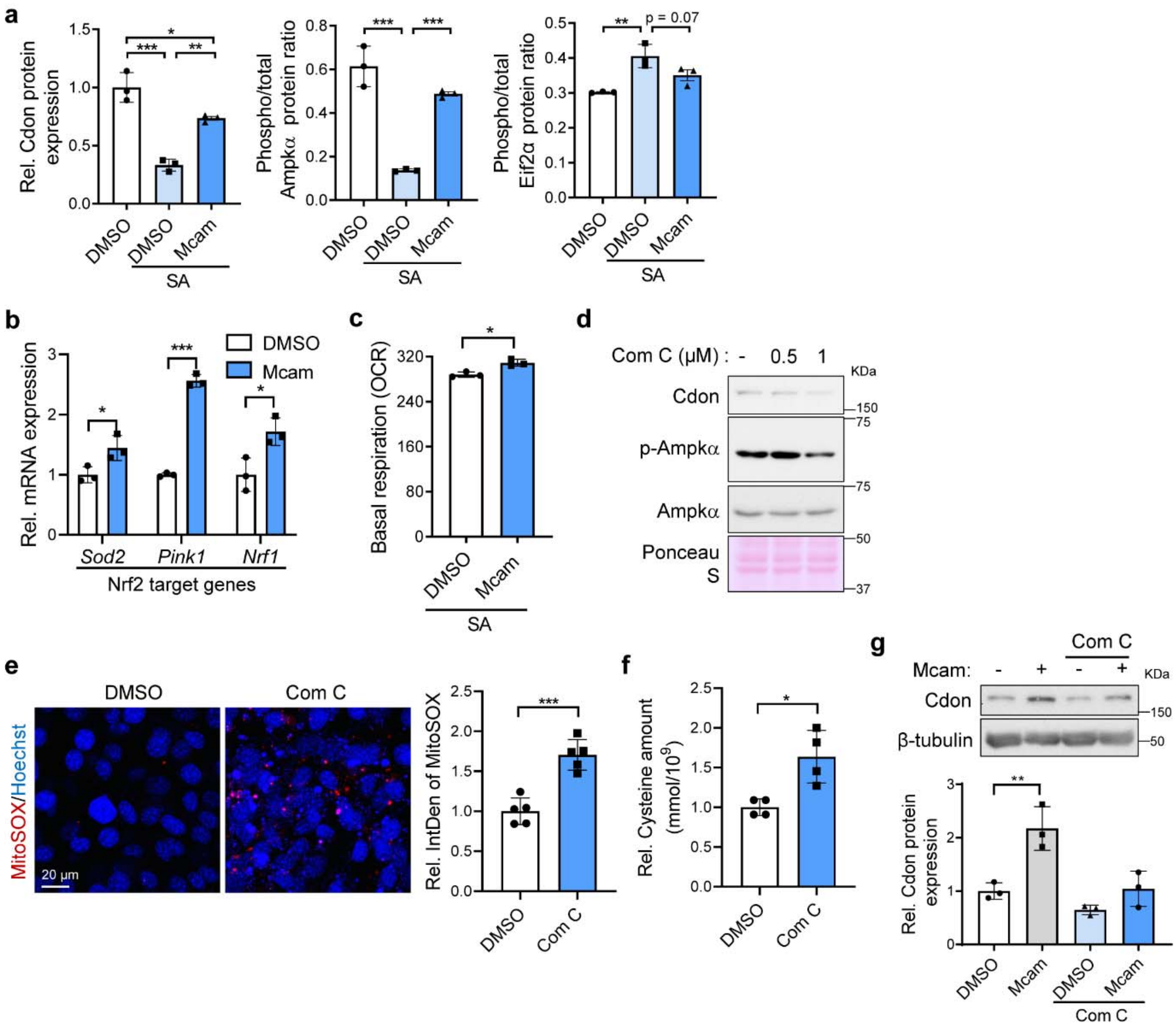

Supplementary Fig. 5: Effects of Mcam on oxidative stress responses and Ampk inhibition–dependent Cdon expression and oxidative stress responsiveness. a Quantification for Cdon protein expression and phosphorylation levels of Ampkα or Eif2α in C2C12 cells, *n* = 3. **b** qRT-PCR analysis for Nrf2 target genes in DMSO- or Mcam-treated C2C12 cells, *n* = 3. **c** Basal respiration (OCR) levels of C2C12 cells. Cells were treated with SA in combination with DMSO, or SA in combination with Mcam for 24 h, *n* = 3. **d** Immunoblot analysis for indicated protein expression in C2C12 cells. Cells were treated with indicated concentration of Com C. **e** Immunoblot analysis for Cdon protein expression in C2C12 cells. Cells were treated with DMSO, Mcam, Com C in combination with DMSO, or Com C in combination with Mcam for 24 h. Below graph indicates Cdon protein expression, *n* = 3. **f** MitoSOX (red) staining in DMSO- or Com C-treated C2C12 cells. Right graph indicates

relative IntDen for MitoSOX, $n = 5$. **g** Relative cysteine amount (mmol/10^9) in DMSO- or Com C-treated C2C12 cells, $n = 4$. Data are presented as mean ± SD. Statistical significance was determined using one-way ANOVA (panel a and g) and unpaired Student's t-test (panel b, c, e and f). *$p < 0.05$, **$p < 0.01$, and ***$p < 0.001$.

Supplementary Table 1. List of used primary antibodies

Antibody	Source	Catalog No.	RRID	Usage
anti-Cdon	Zymed laboratories	51-0800	AB_87627	Immunoblot
anti-PGC-1α	Calbiochem	ST1202	AB_2237237	Immunoblot
anti-Tom20	Abnova	H00009804-M01	AB_519121	IHC
anti-Total OXPHOS	Invitrogen	45-8099	AB_2533835	Immunoblot
anti-MyHC	DSHB (hybridoma)	MF20	AB_2147781	Immunoblot, IHC
anti-MyHC-IIa	DSHB	SC-71	AB_2147165	IHC
anti-MyHC-IIb	DSHB	BF-F3	AB_2266724	IHC
anti-Laminin	Sigma	L9393	AB_477163	IHC
anti-Fast-MyHC	Sigma	M4276	AB_477190	Immunoblot
anti-Slow-MyHC	Sigma	M8421	AB_477248	Immunoblot
anti-p-Akt^{S473}	Cell Signaling Technology	4058	AB_331168	Immunoblot
anti-Akt	Cell Signaling Technology	9272	AB_329827	Immunoblot
anti-p-mTor^{S2448}	Cell Signaling Technology	5536	AB_10691552	Immunoblot
anti-mTor	Cell Signaling Technology	2983	AB_2105622	Immunoblot
anti-p-Creb^{S133}	Cell Signaling Technology	9198	AB_2561044	Immunoblot
anti-Creb	Cell Signaling Technology	9197	AB_331277	Immunoblot
anti-LC3B	Invitrogen	PA1-16930	AB_2281384	IHC
anti-p-Ampkα^{T172}	Cell Signaling Technology	2535	AB_331250	Immunoblot
anti-Ampkα	Cell Signaling Technology	5831	AB_10622186	Immunoblot
anti-Pink1	Santa Cruz	sc-33796	AB_2164259	Immunoblot
anti-Parkin	Santa Cruz	sc-30130	AB_653855	Immunoblot

anti-β-tubulin	Invitrogen	32-2600	AB_2533072	Immunoblot
anti-Baf60c	Cell Signaling Technology	62265	AB_2799624	Immunoblot
anti-MyoD	Novus Biologicals	NBP2-34772	AB_3293191	Immunoblot
anti-Hsp90	StressMarq	SPC-104D	AB_2120930	Immunoblot
anti-phospho-$S6^{S235/236}$ Ribosomal protein	Cell Signaling Technology	2211	AB_331679	Immunoblot
anti-S6 Ribosomal protein	Cell Signaling Technology	2217	AB_331355	Immunoblot
anti-Acc	Cell Signaling Technology	3662	AB_2219400	Immunoblot
anti-Pparγ	Santa Cruz	sc-7273	AB_628115	Immunoblot
anti-Gpx4	Cell Signaling Technology	59735	AB_2940796	Immunoblot
anti-phospho -$Eif2a^{S51}$	Cell Signaling Technology	3398	AB_2096481	Immunoblot
anti-Eif2a	Cell Signaling Technology	5324	AB_10692650	Immunoblot

Supplementary Table 2. List of primer sequences

Human	Sequence	
	**Forward**	**Reverse**
CDON	AGGCTGTGACTCAGGAACATGC	CAGGAAGTGTGACTGTCTCTGC
18s rRNA	GTAACCCGTTGAACCCCATT	CCATCCAATCGGTAGTAGCG
**Mouse**	**Sequence**	
	**Forward**	**Reverse**
Cdon	GTGTGCTTGGGGTTATGGTC	CTCCATTTATGTTTCCACTC
Ki67	GAGGAGAAACGCCAACCAAGAG	TTTGTCCTCGGTGGCGTTATCC
Myh1	TGCAACAGTTCTTCAACCAC	GCCAGGTCCATCCCAAAGT
Myh2	CCAGCTGCACCTTCTCGTTTGCCA	CATGGGGAAGATCTGGTCTTCTTTCACGGTCAC
Myh4	CCTGGAACAGACAGAGAGGAGCAGGAGAG	GTGAGTTCCTTCACTCTGCGCTCGTGC
Myh7	ACAAGCTGCAGCTGAAGGTG	CCAAGCTGCACCTTCTCGTTTGCCAG
Baf60c	CAGGAGATCAGTGCTCTGGACA	AGCAGGTCTTGGACGTAGCCTT
Deptor	AACCATGCCACATCCAGGCTGT	GCGGTAGTCTACATTCAGCACG
Fbxo32	CTTCTCGACTGCCATCCTGGAT	TCTTTTGGGCGATGCCACTCAG
Trim63	TACCAAGCCTGTGGTCATCCTG	ACGGAAACGACCTCCAGACATG
Gpx4	CCTCTGCTGCAAGAGCCTCCC	CTTATCCAGGCAGACCATGTGC
Nrf1	GGCAACAGTAGCCACATTGGCT	GTCTGGATGGTCATTTCACCGC
Nrf2	CAGCATAGAGCAGGACATGGAG	GAACAGCGGTAGTATCAGCCAG
Sod2	TAACGCGCAGATCATGCAGCTG	AGGCTGAAGAGCGACCTGAGTT
Pink1	CGACAACATCCTTGTGGAGTGG	CATTGCCACCACGCTCTACACT
Ppargc1a	ATGTGTCGCCTTCCTTCTTCT	CGGTGTCTGTAGTGGCCTTGA
Ptgs2	GCTCAGCCAGGCAGCAAATC	CACCATAGAATCCAGTTCGGG
Sirt3	GCTACATGCACGGTCTGTCGAA	CAATGTCGGGTTTCACAACGCC
mt-Co1	CTACTATTCGGAGCCTGAGC	GCATGGGCAGTTACGATAAC
18s rRNA	CGGCTACCACATCCAAGGAA	GCTGGAATTACCGCGGCT
mtDNA; *mt-Co2*	ACCTGGTGAACTACGACTGC	GGACTGCTCATGAGTGGAGG
nDNA; *Actb*	CATTGCTGACAGGATGCAGAAGG	TGCTGGAAGGTGGACAGTGAGG
